# Studies of the symmetric binding mode of daclatasvir and analogs using a new homology model of HCV NS5A GT-4a

**DOI:** 10.1007/s00894-022-05420-4

**Published:** 2022-12-29

**Authors:** Kholoud A. Saad, Mohammed A. Eldawy, Khaled M. Elokely

**Affiliations:** 1 Department of Pharmaceutical Chemistry, Tanta University, Tanta, 31527 Egypt; 2 Institute for Computational Molecular Science and Department of Chemistry, Temple University, Philadelphia, PA 19122 USA

**Keywords:** Hepatitis C virus, Direct-acting antivirals, NS5A inhibitors, Molecular docking, Dynamic simulation

## Abstract

**Context:**

Egypt has a high prevalence of the hepatitis C virus (HCV) genotype 4a (GT-4a). Unfortunately, the high resistance it exhibited still was not given the deserved attention in the scientific community. There is currently no consensus on the NS5A binding site because the crystal structure of HCV NS5A has not been resolved. The prediction of the binding modes of direct-acting antivirals (DAA) with the NS5A is a point of controversy due to the fact that several research groups presented different interaction models to elucidate the NS5A binding site. Consequently, a 3D model of HCV NS5A GT-4a was constructed and evaluated using molecular dynamics (MD) simulations. The generated model implies an intriguing new orientation of the AH relative to domain I. Additionally, the probable binding modes of marketed NS5A inhibitors were explored. MD simulations validated the stability of the predicted protein–ligand complexes. The suggested model predicts that daclatasvir and similar drugs bind symmetrically to HCV NS5A GT-4a. This will allow for the development of new NS5A-directed drugs, which may result in reduced resistance and/or a wider range of effectiveness against HCV.

**Methods:**

The 3D model of HCV NS5A GT-4a was constructed using the comparative modeling approach of the web-based application Robetta. Its stability was tested with 200-ns MD simulations using the Desmond package of Schrodinger. The OPLS2005 force field was assigned for minimization, and the RMSD, RMSF, and rGyr were tracked throughout the MD simulations. Fpocket was used to identify druggable protein pockets (cavities) over the simulation trajectories. The binding modes of marketed NS5A inhibitors were then generated and refined with the aid of docking predictions made by FRED and AutoDock Vina. The stability of these drugs in complex with GT-4a was investigated by using energetic and structural analyses over MD simulations. The Prime MM-GBSA (molecular mechanics/generalized Born surface area) method was used as a validation tool after the docking stage and for the averaged clusters after the MD simulation stage. We utilized PyMOL and VMD to visualize the data.

**Supplementary information:**

The online version contains supplementary material available at 10.1007/s00894-022-05420-4.

## Introduction

**Fig. 1:**
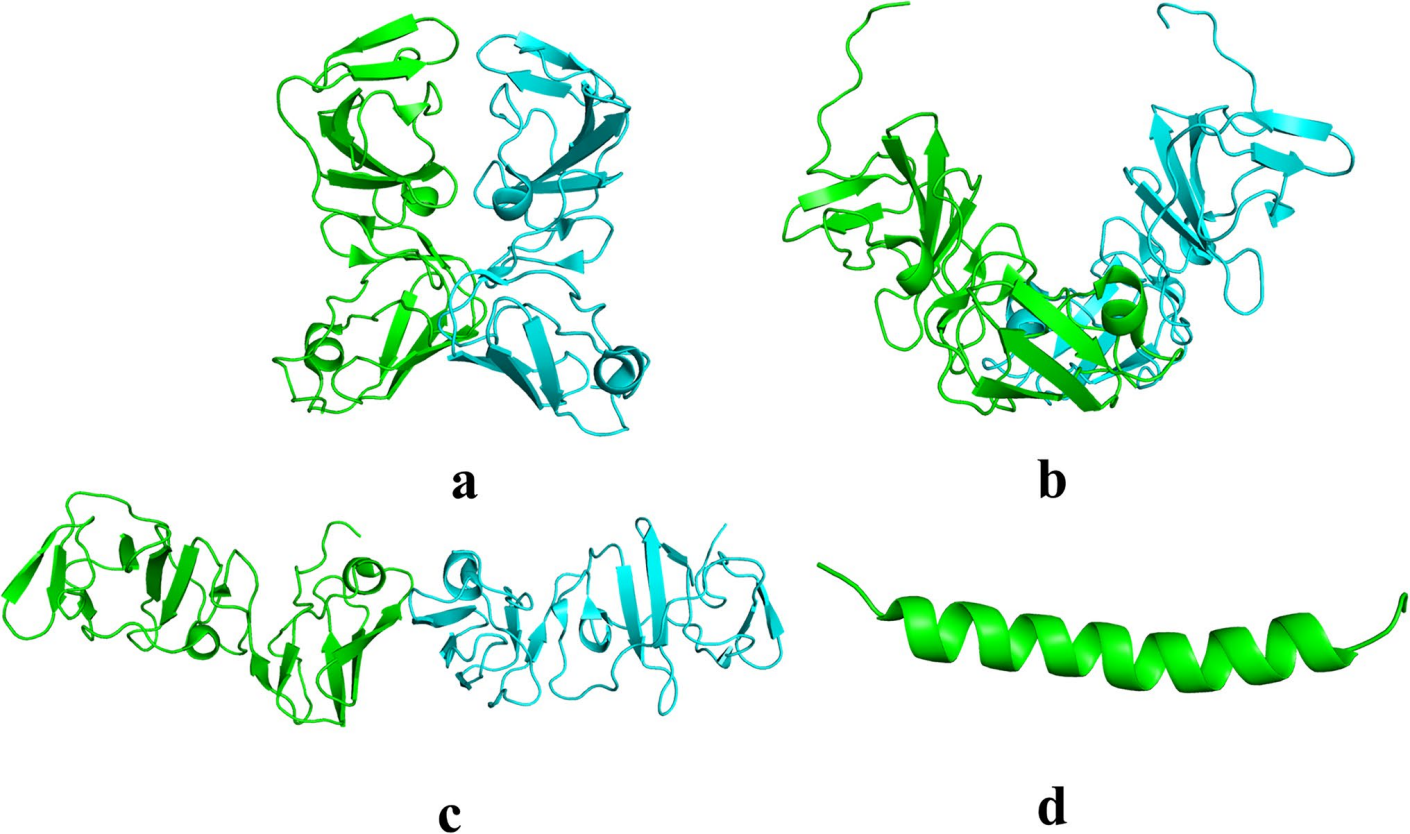
The four solved D-Ia structural fragments (**a**) PDB code: 1ZH1 residues 36 − 198 [8], (**b**) and (**c**) PDB codes: 3FQQ and 4CL1 residues 32 − 191 [10, 11], (**d**) PDB code: 1R7C residues 1 − 31 [12]. Figure generated using PyMOL [13]

More than 170 million people around the world have chronic infections due to the hepatitis C virus (HCV), making it a major public health concern and earning it the label “silent epidemic.” [1]. In Egypt, GT-4a dominates the HCV epidemic [2]. There is no preventive vaccine against HCV, but research in this field is ongoing. Until recently, ribavirin and interferon-α were the therapy standards. Nonetheless, due to undesirable side effects, researchers have developed more effective medicines targeting non-structural (NS) NS3/4 protease, NS5A D-I, and NS5B polymerase activities, which could cure the infection in approximately 98% of cases [3]. The NS5A protein is 447 amino acids long and is structurally split into three main domains. It is believed that D-I and possibly D-II are essential for viral replication, while D-III is needed for viral packaging and release [4, 5]. D-I is the only intrinsically structured domain, and it is distinguished by its α-helix (AH), which is thought to connect the NS5A protein to the endoplasmic reticulum and lipid droplet [6, 7], and cytosolic subdomains (D-Ia and D-Ib). The active site region of NS5A cannot be accurately modeled because its enzymatic activity is not known [8, 9]. Figure 1 illustrates the four D-I structural fragments. D-I crystal structure (PDB: 1ZH1) monomers included residues 36–198 in an “open conformation.” Although one of the 3FQQ monomers is missing the L32 amino acid residue, the D-I crystal structures (PDB codes: 3FQQ “closed conformation” and 4CL1) covered residues 32 through 191 [8, 10, 11]. The N-terminal AH (PDB code: 1R7C) spanned residues 1 through 31 [12]. The flexible AH-D-I linker is made up of residues 26–33.

Seven groups provide distinct interaction models to understand the NS5A binding site [10, 14–19], and the resulting 3D structure of NS5A in complex with direct-acting antivirals (DAA) remains controversial. In this study, we used molecular modeling techniques to generate a novel and robust homology model for HCV NS5A GT-4a, which includes the N-terminal region of NS5A encompassing the AH and D-I, the site of numerous resistance mutations. To add intrigue, the generated model reveals a novel orientation of the AH with respect to D-I and may be used to develop and evaluate novel antiviral drugs that target the NS5A protein. The binding region of daclatasvir and analogs to HCV NS5A GT-4a is also described in this work.

## Materials and methods

### Homology modeling

A full-length model of NS5A D-I was proposed using homology modeling; this model includes the AH, the linker domain, and the cytosolic subdomain (Fig. S1).

The UniProt database was accessed for the HCV GT-1a NS5A sequences [20] (entry and position: P27958 [1973—2163]). The 3D model of HCV GT-1a was built using the comparative modeling technique of the web-based application Robetta [21], utilizing the crystal structure of the cytosolic subdomain (PDB code: 3FQQ) as a template. The constructed model monomer was used as a building block in the PyMOL molecular graphics engine to create the homodimer [13]. Assembly of the homodimer was guided by the crystal structure (PDB code: 3FQQ). The protonation state of the titratable residues of the studied targets was calculated using the empirical program PropKa [22]. The YASARA energy minimization server [23] was then used to further relax the modeled 3D structure of HCV NS5A GT-1a. Finally, PROCHECK and Verify3D [24, 25] were used to verify the energy-optimized model.

A brief summary of the minimization protocol: first, the server performs about 40 different cleanup operations to get the structure ready for simulation; then, using the SCWRL algorithm, it predicts the best amino acid side-chain rotamers; finally, it uses the YASARA2 force field with implicit solvent to fine-tune the rotamers via steepest descent minimization in dihedral angle space. Then, the protonation states and side-chain pK are optimized for a pH of 7.4, and the solute is encased in an explicit solvent shell of 6 Å thick. Next, the steepest descent minimization was executed to clear away any residual collisions, and finally, a simulated annealing minimization was employed, decreasing the atoms’ velocities by a factor of 0.9 every 10 steps until a stable local minimum in energy was found. Adjustments for inaccurate side-chain isomers and cis-peptide linkages are made during minimization. After 200 cycles, if the energy has decreased by less than 0.05 kJ/mol per atom, the minimization process is terminated. Energy levels are shown as the sum of force field energy and implicit solvation energy. The displayed quality Z-scores are arithmetical averages of the “Dihedrals,” “Packing1D,” and “Packing3D” values [23].

### Molecular docking and binding energy determination

ChemAxon® MarvinSketch 5.1.1.5 [26] was used to build the three-dimensional structures of the studied compounds (daclatasvir, ledipasvir, ombitasvir, elbasvir, velpatasvir, and pibrentasvir). OMEGA 2.5.1.4 [27] (OpenEye) was used as the conformer generator. The protonation state of the scrutinized compounds was investigated using fixpka application implemented within QUACPAC software [28]. The Make Receptor utility from OpenEye [29] was used to create the receptor grid for docking. A 3D box enclosing the main active site was generated based on the center of selected amino acids known to interact with the reported NS5A inhibitors [10]. The FRED docking protocol [30, 31] of OEDocking v3.2.0.2 was utilized to dock the prepared ligands into HCV NS5A GT-1a and GT-4a models. Ten poses for each compound were generated.

AutoDock Vina [32, 33] integrated into UCSF Chimera (version 1.13.1) [34] was used to conduct a molecular docking investigation, the findings of which were compared to those obtained using OEDocking. All nonstandard atoms and bonds were removed from the protein’s PDB structure using UCSF Chimera version 1.13.1 [34]. After loading this protein into AutoDock Vina, the grid box function was used to pinpoint its binding pocket’s precise coordinates. The grid box size was set to 46.8738 × 21.9098 × 25.6067 Å with a grid box center of 24.2825 × 6.38342 × 25.2645 Å. Using the dock prep feature, we removed water molecules, set partial charges, and incorporated hydrogen atoms into the protein. After that, the protein was saved in mol2 format for the docking step to begin. The investigated compounds were sketched, optimized, and stored in mol2 format utilizing ChemAxon® MarvinSketch 5.1.1.5 [26]. The “structure editing function” was then used to include hydrogen atoms into the structure, and the “minimize structure tool” was implemented to drive the system toward energy minimization. In order to get ready for the docking phase, we named and saved the ligand in the mol2 format. The protein mol2 file was loaded into a separate session of UCSF Chimera, and the target molecule was loaded afterward. The AutoDock Vina procedure was completed using the surface/binding analysis instrument. It was assumed that the protein PDB format would serve as the receptor, and that the target molecule SMILES would serve as the ligand. The coordinates of the binding pocket were written into an output. Exhaustiveness of the search was set to a maximum of 8, and all ligand and receptor possibilities were marked as true. Ten poses for each compound were generated.

As a post-docking validation tool and for the averaged clusters following the molecular dynamics (MD) simulation stage (described later), the Prime MM-GBSA (molecular mechanics/generalized Born surface area) method was utilized. To do this, the following equation is used to determine the binding free energy (∆*G*_bind_) of each ligand based on the protein–ligand complex:

$$\Delta {G}_{\text{bind}}=\Delta {E}_{\text{MM}}+\Delta {G}_{\text{solv}}+\Delta {G}_{\text{SA}}$$

where ∆*E*_MM_ is the difference in energy between the complex structure and the sum of the energies of the protein with and without ligand, ∆*G*_solv_ is the difference in the GBSA solvation energy of the complex and the sum of the solvation energies for the ligand and unliganded protein, and ∆*G*_SA_ is the difference in the surface area energy for the complex and the sum of the surface area energies for the ligand and uncomplexed protein. As the MM/GBSA binding energies are approximate free energies of binding, a more negative value indicates stronger binding.

### Dynamic simulation

To evaluate the stability of the modeled protein and confirm the binding affinity of the ligands, we projected the modeled HCV NS5A GT-4a protein (apoprotein) and protein–ligand complexes (obtained by docking) to triplicate MD simulation runs using the Desmond module of Schrödinger [35, 36]. Each system was solvated using the TIP3P [37] water model within an orthorhombic box shape. The systems were then neutralized with Na^+^ ions, and the OPLS2005 force field was assigned for minimization. The apoprotein and protein–ligand complexes were immersed in the POPC lipid bilayer. These systems were evaluated in an NPT (normal pressure and temperature) environment. Desmond’s default protocols [35, 36] were carried out to achieve system minimization and equilibrium. Both apoprotein and protein–ligand complexes were simulated for a constant 200 ns. The SHAKE algorithm was used to constrain all hydrogen-containing bonds. The particle mesh Ewald (PME) technique was used to account for the effects of Coulombic interactions across long ranges. For the production stage’s post-dynamics analysis and binding energy estimates, the trajectories were saved every 10 ps. The trajectories were investigated using a variety of metrics, including the distance between the center of the mass of the AH and the center of the membrane (COM), protein pocket (cavity) identification using Fpocket [35], the root mean square deviation (RMSD) of the protein backbones, the root mean square fluctuation (RMSF), and the radius of gyration (rGyr). The interaction intensity was computed after each MD simulation run by measuring the frequency of occurrences in the trajectory utilizing the simulation interaction diagram. N.B. Before the docking study, an MD simulation of the apoprotein was carried out to ensure the stability of the generated model. MM-GBSA calculation was carried out over the MD simulation trajectories.

#### Figure generation

Images were generated using PyMOL [38] and VMD [38] visualization tools, and the graphs were plotted using the Grace program [39].

## Results and discussion

### Homology modeling

**Fig. 2:**
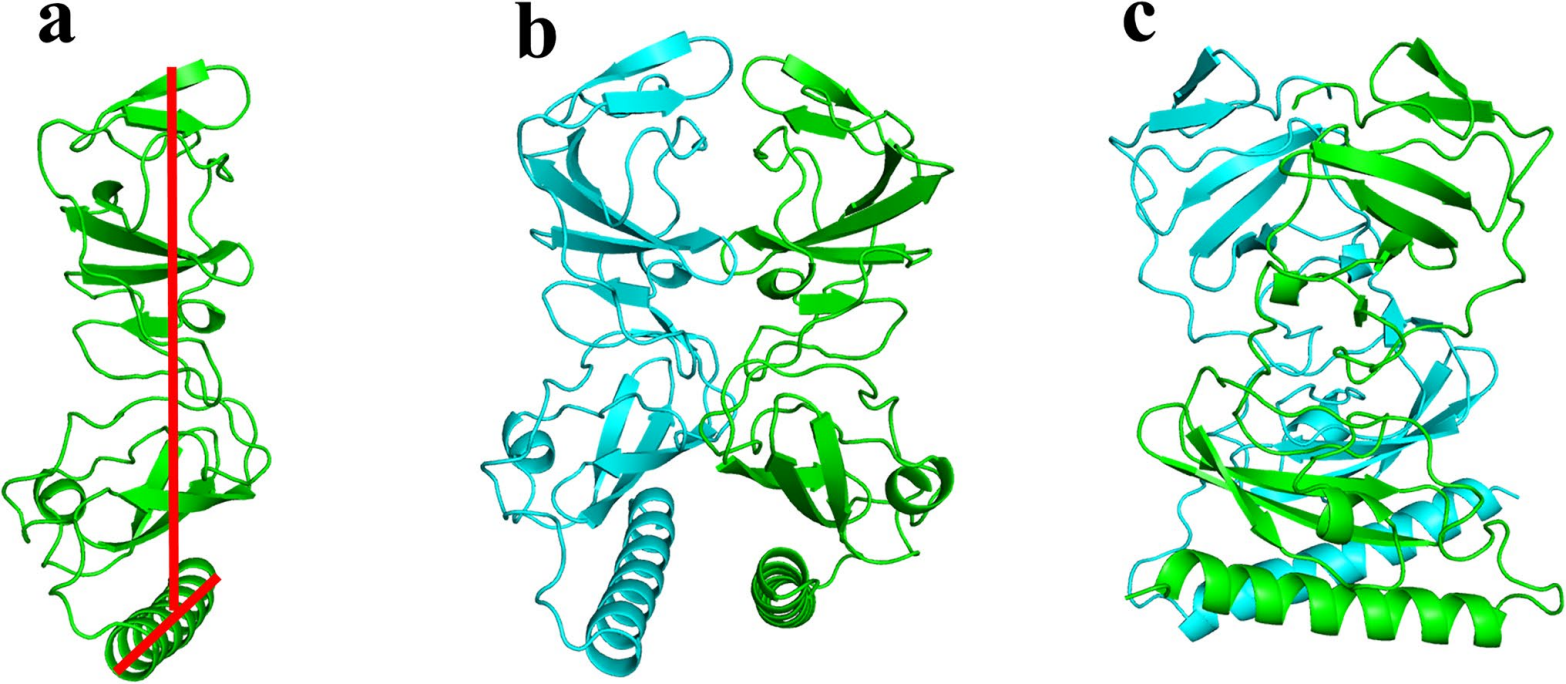
HCV NS5A GT-1a model using Robetta server [19]. (**a**) A monomer of NS5A GT-1a. (**b**) A homodimer was constructed using 3FQQ as a template. (**c**) A side view of the homology-modeled structure. The figure was generated using PyMOL [13]

Using the comparative modeling technique of the Robetta online tool, a homology model covering the entire length of HCV NS5A GT-1a was generated. The GT-1a sequence was acquired from the UniProt database [20]. PyMOL [13] was utilized to build the homodimer from the derived monomeric model (Fig. 2). To improve the accuracy of the model, energy minimization was carried out utilizing the YASARA energy minimization server. The Ramachandran plot from PROCHECK was used to verify the model accuracy. The results showed that, out of 382 residues, 284 (91.6%) are in the most preferred regions, and 26 (8.4%) are in the additional permitted region. None of the residues was in the plot area that was not allowed (Fig. S2).

**Fig. 3:**
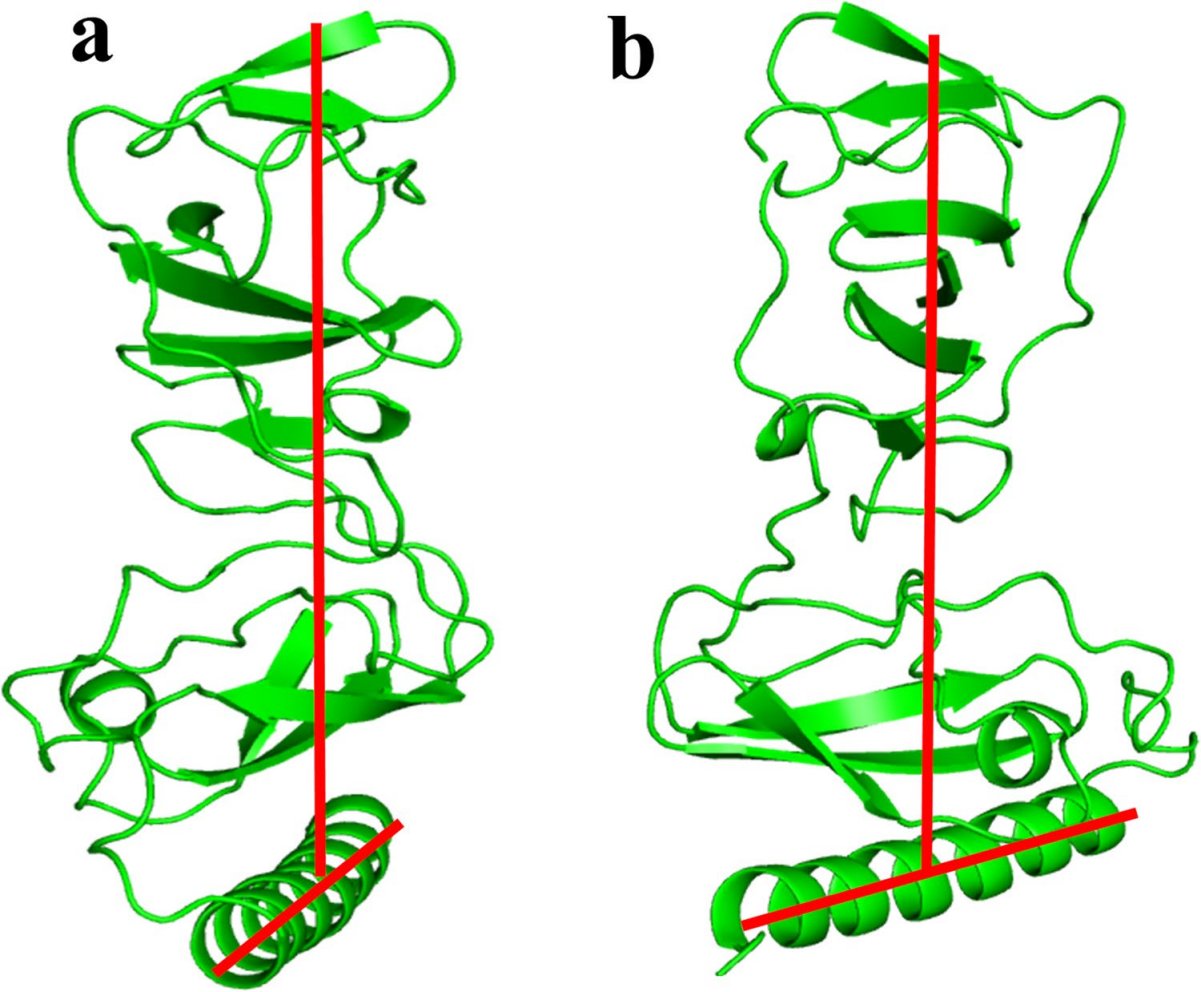
T-shaped orientation of AH (**a**) front view and (**b**) side view. The figure was generated using PyMOL [13]

This model uncovered a hitherto undiscovered orientation of AH with respect to the cytosolic subdomain, with the AH axis almost perpendicular to the cytosolic subdomain axis (T-shaped) (Fig. 3).

The GT-1a model was then used as a template on the Robetta server to build the HCV NS5A GT-4a model, which was relaxed via energy minimization (Fig. S3). The Ramachandran plot was used to assess the model, and the results showed that out of 382 residues, there were 286 (92.9%) residues in the most favored regions, 20 (6.5%) residues in the additional allowed region, and two residues (0.6%) were present in the disallowed regions. This validated the quality of the resulting models (Fig. S4).

The average 3D-1D score of the residues in GT-1a (Fig. S5) and GT-4a (Fig. S6) from the verify 3D analysis [25] was > 0.2 for 91.36% and 86.91%, respectively. Based on the assumption that the two models are consistent with their sequence, Verify3D recommends that a good model has at least 80% of the amino acids with a score of 0.2 in the 3D-1D profile. Therefore, based on the Ramachandran plot and Verify 3D analysis, the proposed structures could be regarded as suitable and acceptable models.

### Docking computations of NS5A inhibitors

**Fig. 4:**
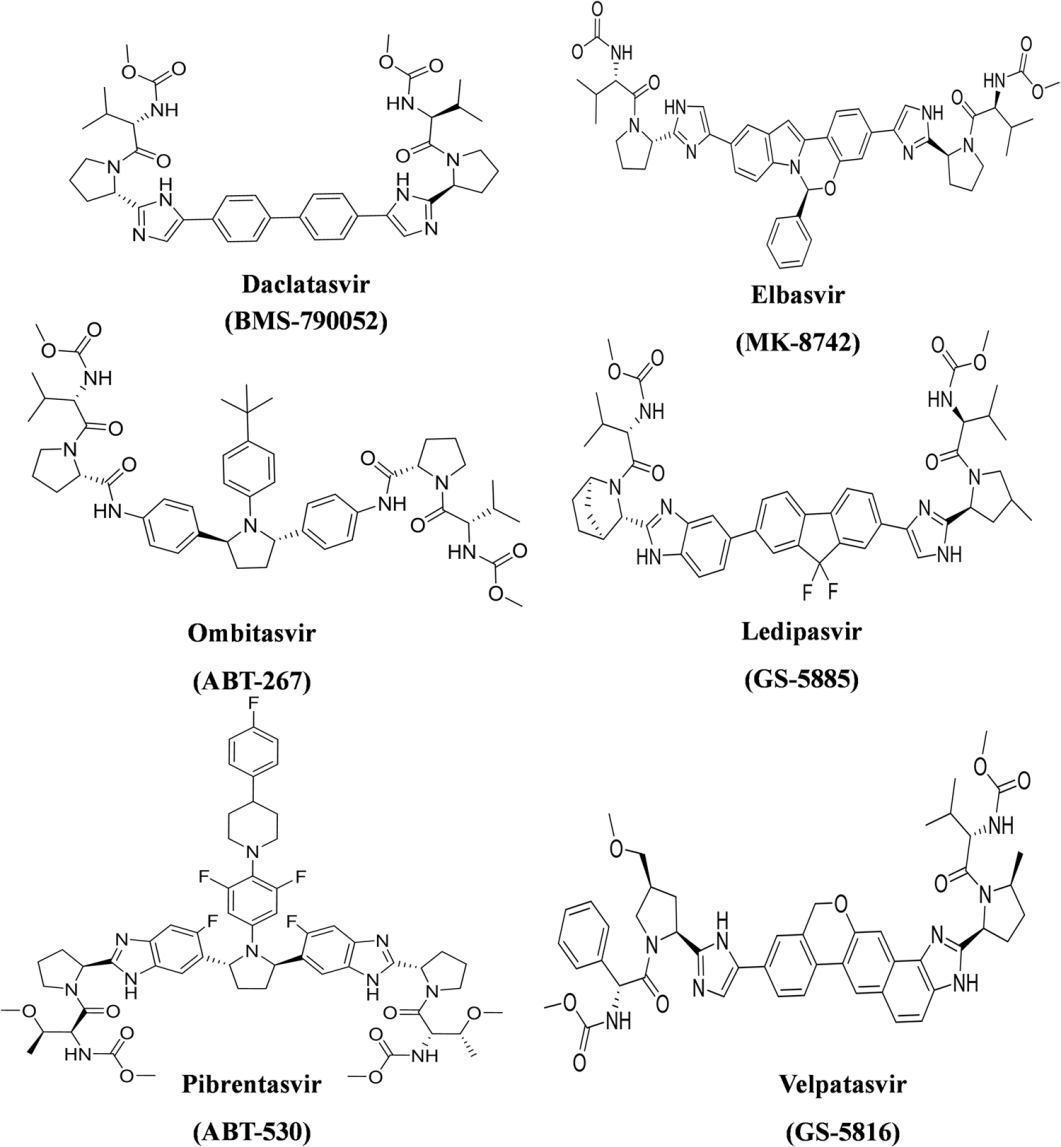
Structures of the studied NS5A inhibitors

The purpose of the current docking study was to define the binding modes and to explore the variations in the interaction of the studied NS5A inhibitors (Fig. 4) with their target (HCV NS5A GT-4a) and try to correlate with their relative potency.

**Fig. 5:**
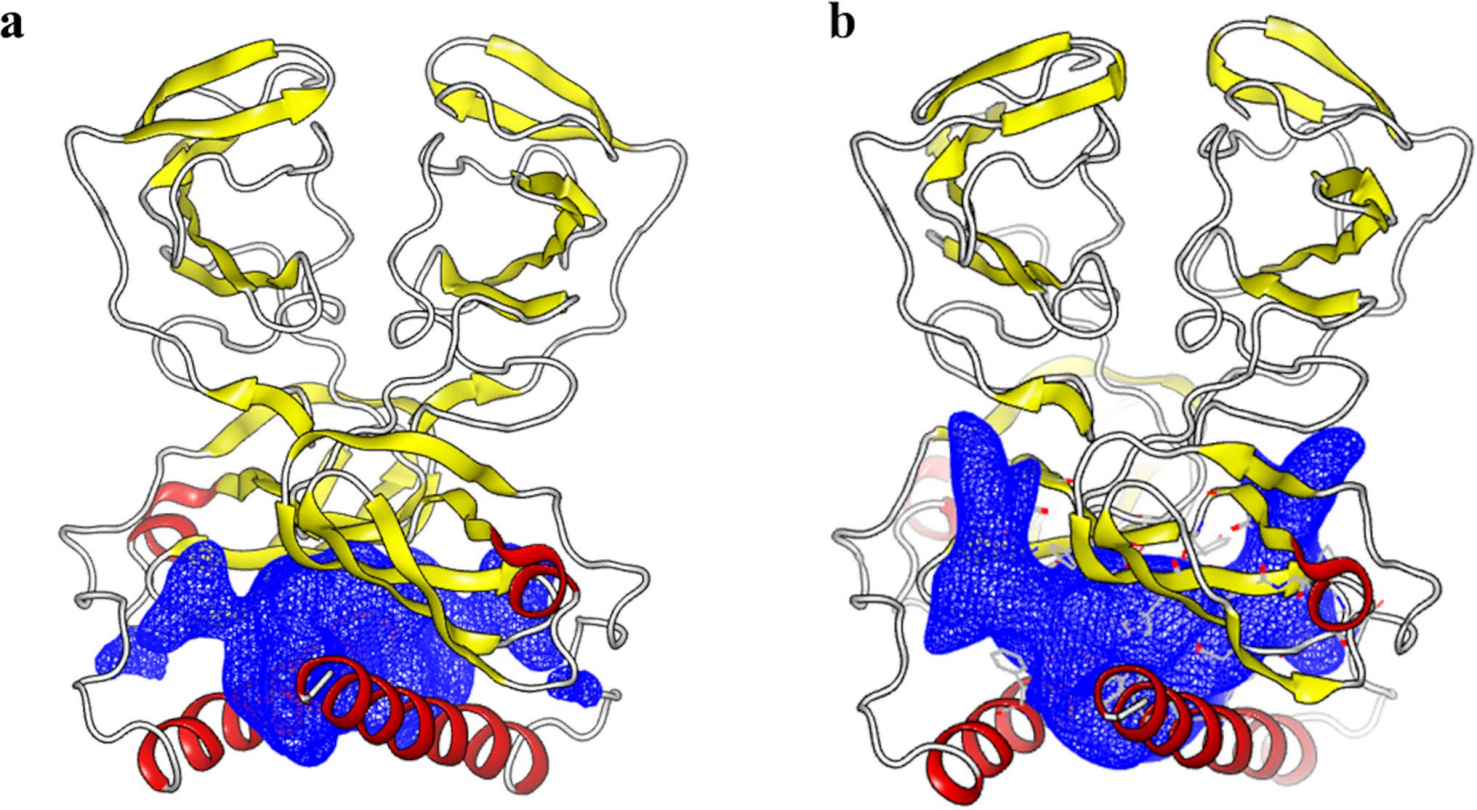
HCV NS5A defined binding sites for GT-1a (**a**) and GT-4a (**b**)

The symmetrical cavity of the NS5A GT-1a and GT-4a models was identified using pocket analysis as the druggable binding site (Fig. 5). The AH surrounds this cavity on both sides, which is close to where the dimer links to the membrane.

For the most part, NS5A inhibitors have three main substructures. Two-terminal peptide caps of varying sizes and characters surround a central hydrophobic aromatic biaryl core, with most caps including an amide group residue (Fig. S7).

When docked into HCV GT-1a and GT-4a, daclatasvir and its analogs were found to bind symmetrically to the NS5A dimer (Fig. S8). The symmetric binding mode is supported by the symmetric features of the ligand and the binding site. In this context, symmetry does not refer to the molecular structure, but rather to the pharmacophore. When comparing the anticipated and observed binding affinities, MM-GBSA binding energy calculations reveal a relatively acceptable agreement (Table 1).

**Table 1:** Docking scores of the compounds under study and their calculated MM/GBSA binding energies

#	Name	HCV NS5A GT-1a			HCV NS5A GT-4a		
		Chemgauss4 scores	EC_50_ (nM) [40]	MM/GBSA Δ*G*_bind_ (kcal/mol)	Chemgauss4 scores	EC_50_ (nM) [40]	MM/GBSA Δ*G*_bind_ (kcal/mol)
1	Daclatasvir (BMS-790052)	− 10.56	0.06	− 75.29	− 11.9	0.03	− 113.97
2	Elbasvir (MK-8742)	− 8.88	0.007	− 104.86	− 9.9	0.006	− 114.81
3	Ledipasvir (GS-5885)	− 12.9	0.01	− 91.56	− 11.3	0.06	− 73.41
4	Pibrentasvir (ABT-530)	− 13.2	0.006	− 105.35	− 6.02	0.004	− 117.46
5	Ombitasvir (ABT-267)	− 7.22	0.01	− 90.68	− 7.91	0.001	− 128.95
6	Velpatasvir (GS-5816)	− 9.2	0.02	− 80.48	− 9.48	0.01	− 114.12

The inhibitors bind similarly to GT-1a and GT-4a, with the biaryl core extended above the central region of the pocket and the two terminal caps interacting with surrounding residues via H-bonds with Arg56 in GT-1a (Fig. S9) and Thr56 in GT-4a (Fig. S10). In vitro replicon experiments show that Daclatasvir is active against both GT-1a and GT-4a, with EC50 (nM) values of 0.03 and 0.06, respectively [40]. Because Arg56 is bigger and positively charged compared to Thr56, steric conflict arises between daclatasvir and the bulky guanidinium component of the Arg56, preventing daclatasvir from penetrating the NS5A dimer interface cleft to the same extent as in the case of GT-4a (Fig. S11).

**Fig. 6:**
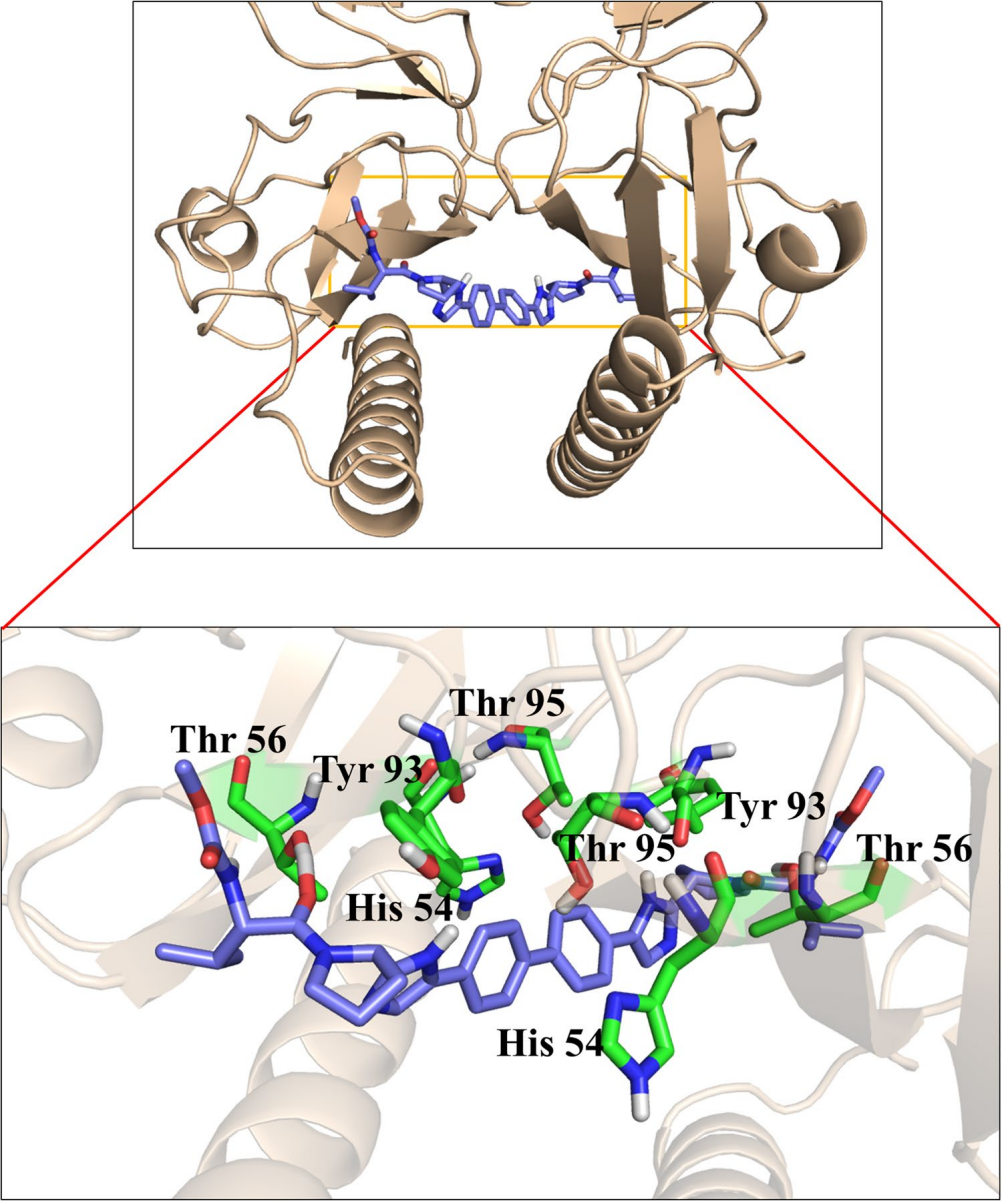
Magnified view of daclatasvir binding to HCV NS5A GT-4a. The figure was generated using PyMOL [13]

Specifically, the binding mode analysis of daclatasvir to HCV NS5A GT-4a showed that the biaryl core fits into a hydrophobic pocket formed by His54, Thr95, and Tyr93 at the central dimer interface. As a result, the biphenyl rings are positioned atop the two methyl groups of Thr95, with the His54 residues of each monomer forming π-π stacking contacts with them. The phenyl rings of the two Tyr93 residues stack with the two imidazole rings of daclatasvir, forming π-π contacts. Trp11, Val15, and Phe19 in the AH complete the hydrophobic pocket. Similarly, the two side peptide caps form H-bond contacts with the side chains of Thr56 and hydrophobic interactions with Pro58 and Pro32 on each side of each monomer (Fig. 6).

Analogs of daclatasvir have demonstrated similar patterns of interaction. All the ligands, including those that differ structurally from daclatasvir (e.g., ledipasvir, velpatasvir, and pibrentasvir), bind symmetrically to the target pocket. The differences between the ligands include different fused rings in place of the biphenyl core and varying lengths of the core and the two wings. The next section describes the compounds that differ significantly from daclatasvir yet have the same two caps; these compounds differ in the central structure, and the effect of this difference was examined in detail.

Daclatasvir has a biphenyl core, but elbasvir has a phenyl ring attached to a fused tetracyclic indole core. This linear structural arrangement on the core makes the compound stiffer than daclatasvir, and its in vitro activity against NS5A is more potent as a result. As was predicted for daclatasvir, the imidazole group retained the π-π stacking contacts with the Tyr93 residue, and a new π-π stacking interaction was created with Phe19 in the helix (Fig. S12). Because of its proximity to the membrane, the phenyl ring made additional AH contact (Fig. S13). Ombitasvir has a bulky core tri phenyl pyrrolidine center and did not produce π-π stacking as daclatasvir did (Fig. S14). Ombitasvir, unlike many other inhibitors, exhibits a somewhat skewed confirmation inside the binding site.

In several NS5A inhibitors, alterations to the central structural region and both caps have increased their antiviral efficacy against a broad spectrum of genotypes and boosted their pharmacokinetics features, such as extending the half-life [14]. Ledipasvir has a bulkier central aromatic core (difluoro-fluorene) and a distal azabicyclic ring. The most appropriate binding mode of ledipasvir is illustrated in Fig. S15. As with daclatasvir, the drug retains all-important interactions with NS5A. The azabicyclic ring in the cap segment of the molecule also allows ledipasvir to interact with the Pro32 residue. Substituting the difluoro-fluorene ring for the biphenyl core tends to increase the overall rigidity of the molecule, allowing ledipasvir to form an H-bond with the side chain of Thr95. In addition, fluorine has an impact on the PK half-life [41].

**Fig. 7:**
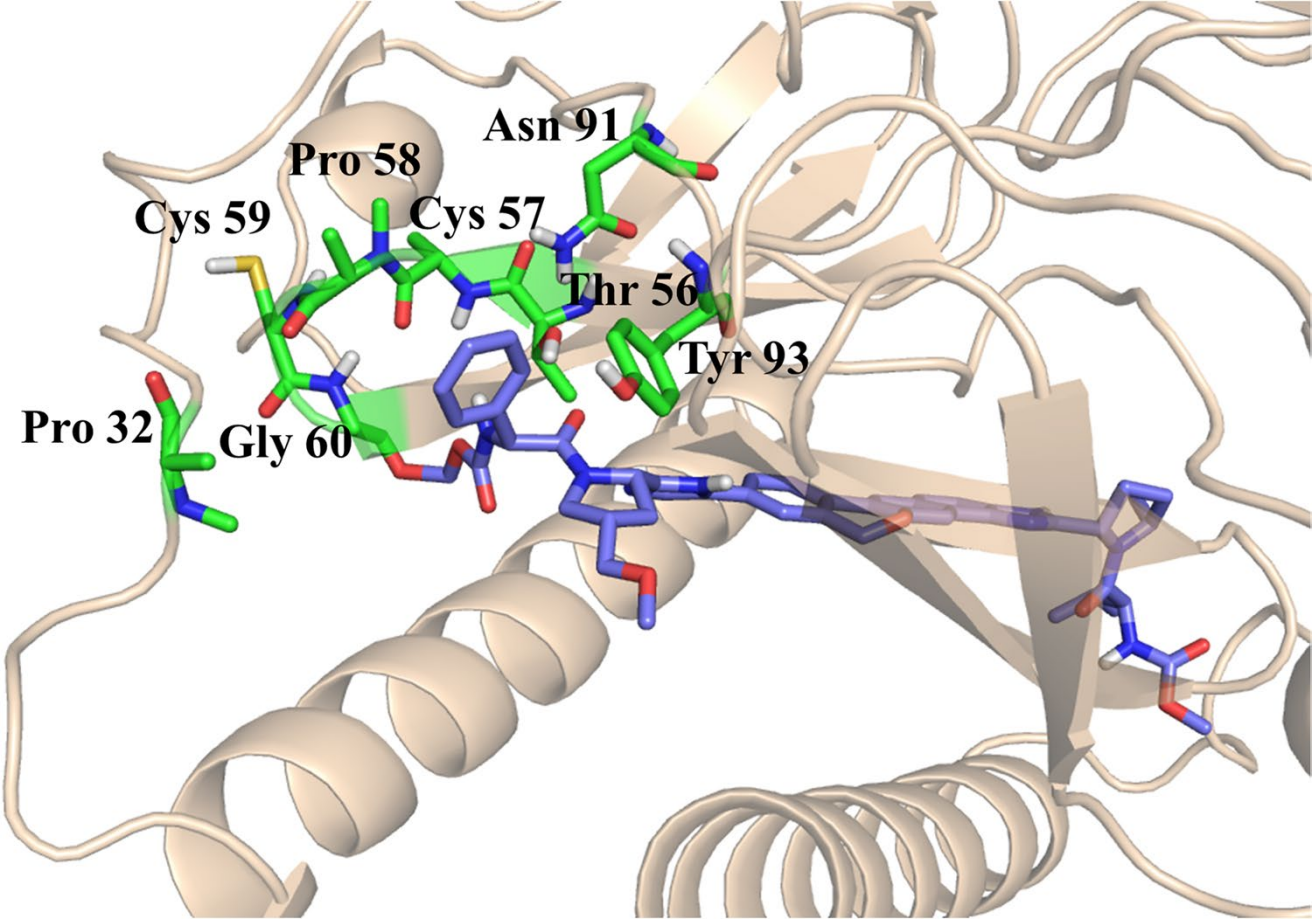
NS5A dimer complexed with velpatasvir, where the phenyl group has a deep shielding

The structures of velpatasvir and elbasvir are similar. However, the core in velpatasvir is slightly extended with a pentacyclic spacer instead of a tetracyclic one leading to increased rigidity. Moreover, velpatasvir introduces asymmetry on the two caps by substituting one of them with methoxymethyl and phenyl glycine. The pentacyclic core leads to an H-bond between Thr56A and the imidazole ring, while the cap maintains the H-bonds with the side chains of Thr56B (Fig. S16). Being an asymmetric compound, velpatasvir had a bit different interaction. The phenyl ring in the cap diffuses significantly into the pocket formed by residues pro32, Pro58, gly60, Asn91, and Pro97 (Fig. 7).

With respect to pibrentasvir, the bis-anilide structure in ombitasvir has been modified to fluorinated bis-benzimidazole (Fig. S17). This modification, combined with inverting the stereochemical link at the pyrrolidine central core and increasing the size and lipophilicity of the anchor group, enables pibrentasvir to interact with more residues in the helix. Incorporating the methoxy group in the peptide cap allows the pibrentasvir to interact with Pro32. Otherwise, the drug has the same essential interactions with NS5A as daclatasvir (Fig. S18).

The supplementary material includes a table of all docking poses and scores of the compounds evaluated by OEDocking and AutoDock Vina (Table S1). The binding modes are substantially conserved and exhibit slight orientation changes, as seen in Fig. S19.

### Molecular dynamics simulation analysis

**Fig. 8:**
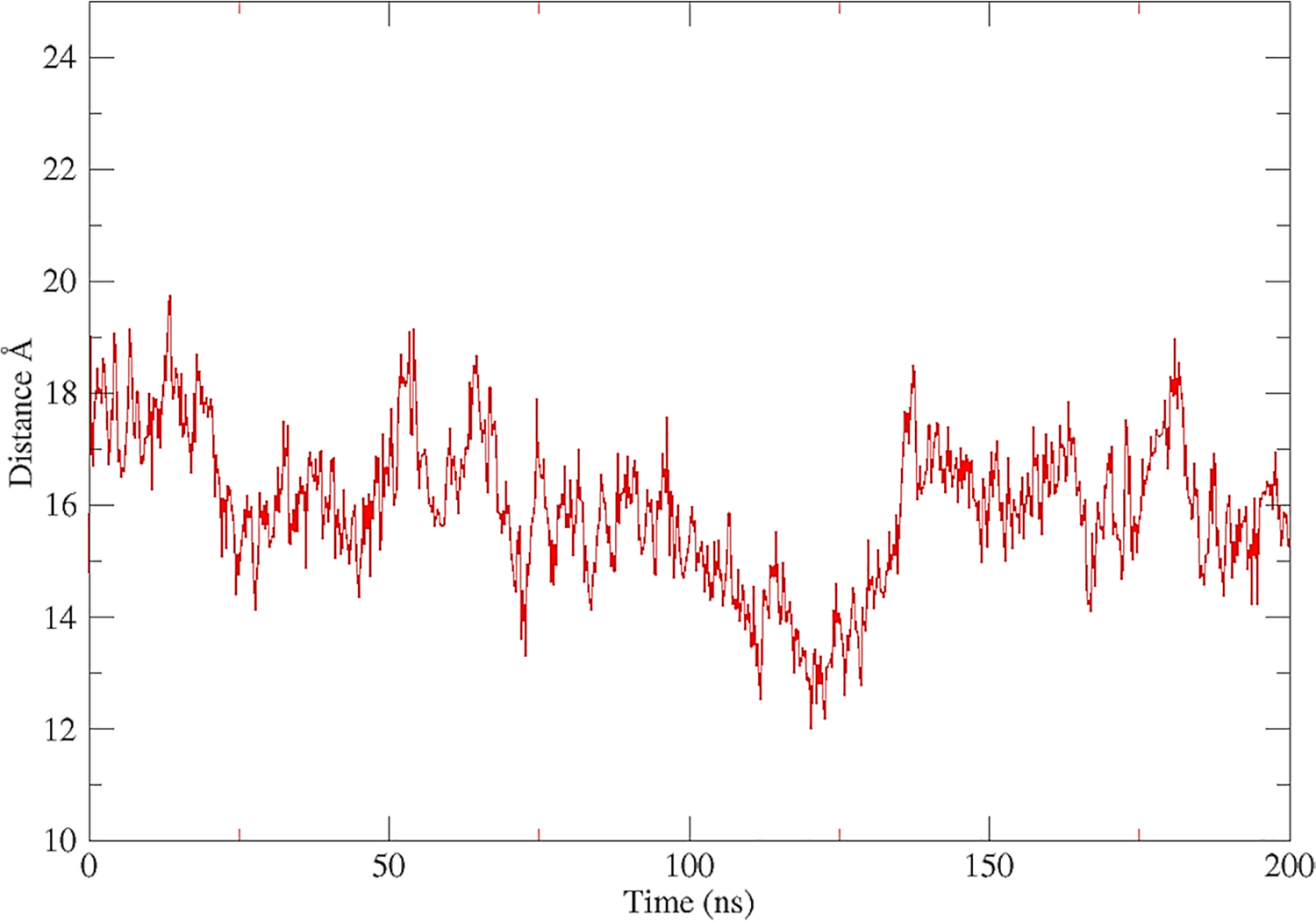
The distance between the center of mass of AH and the center of the membrane during 200-ns MD simulation

To assess the modeled protein and the stability of the protein–ligand complexes (ligands: daclatasvir, ledipasvir, ombitasvir, elbasvir, velpatasvir, and pibrentasvir), MD simulations of the apoprotein and complexes were conducted for 200 ns. Analysis of the MD simulation of the apoprotein showed that the distance between the center of the mass of the AH and the center of the membrane (COM) ranges within ~ 4 Å throughout the 200-ns MD simulation, and it appears that the AH is submerged in the membrane between 100 and 150 ns then relocated to its primary position as a membrane anchor domain (Fig. 8 and Fig. S19).

**Fig. 9:**
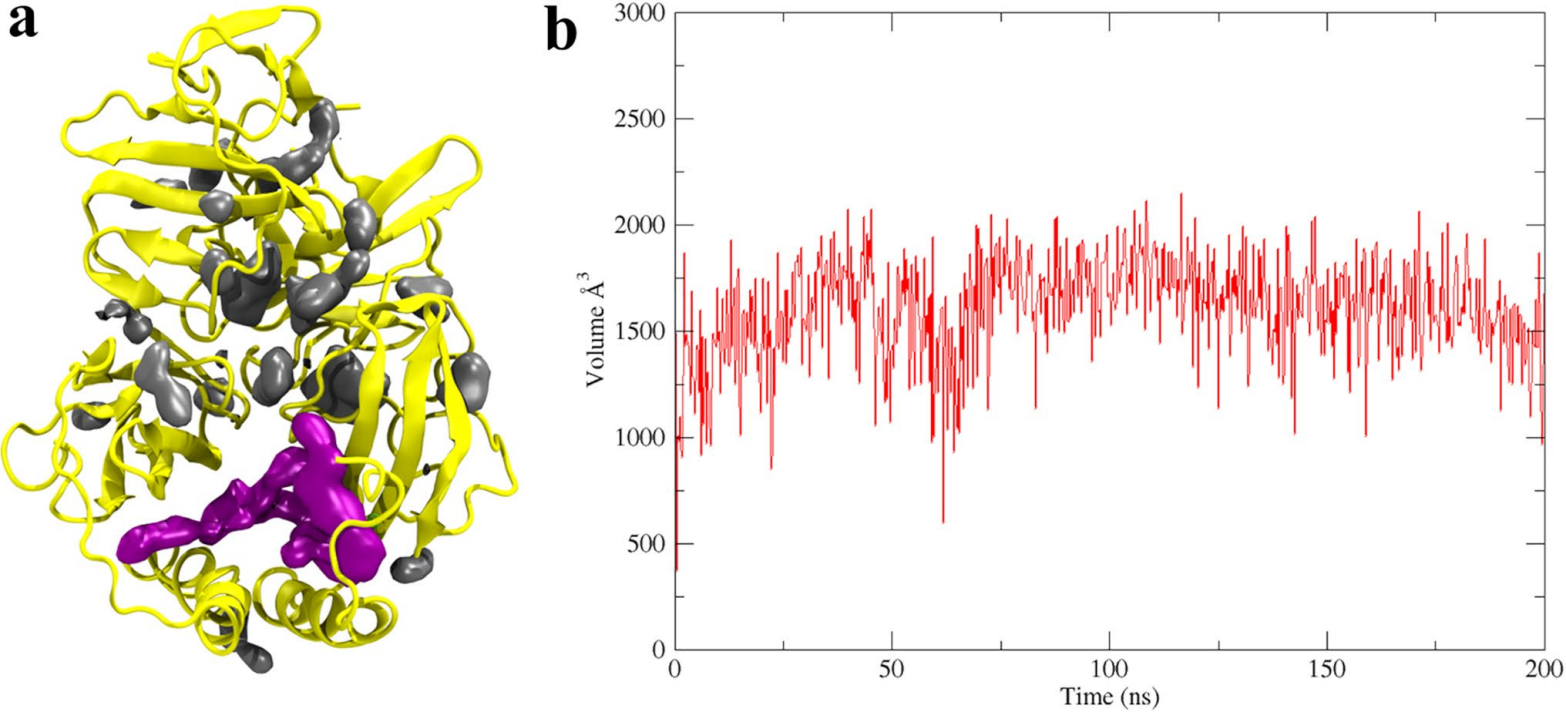
(**a**) Detected pockets using Fpocket. (**b**) The volume of the binding pocket during 200-ns trajectory

Fpocket [42] was used for the detection of druggable protein pockets (cavities). The largest (magenta-colored) pocket was determined to be the binding site because of its high druggability score. It also contained the amino acids predicted to interact with the known NS5A inhibitors [10]. Mdpocket [42] was then used to track the pocket during MD simulations. The plot of the Mdpocket shows that the binding pocket volume fluctuates between 1000 and 2000 Å^3^ giving a stable cavity with a volume of ~ 500 Å^3^ throughout the 200-ns MD simulations (Fig. 9).

**Fig. 10:**
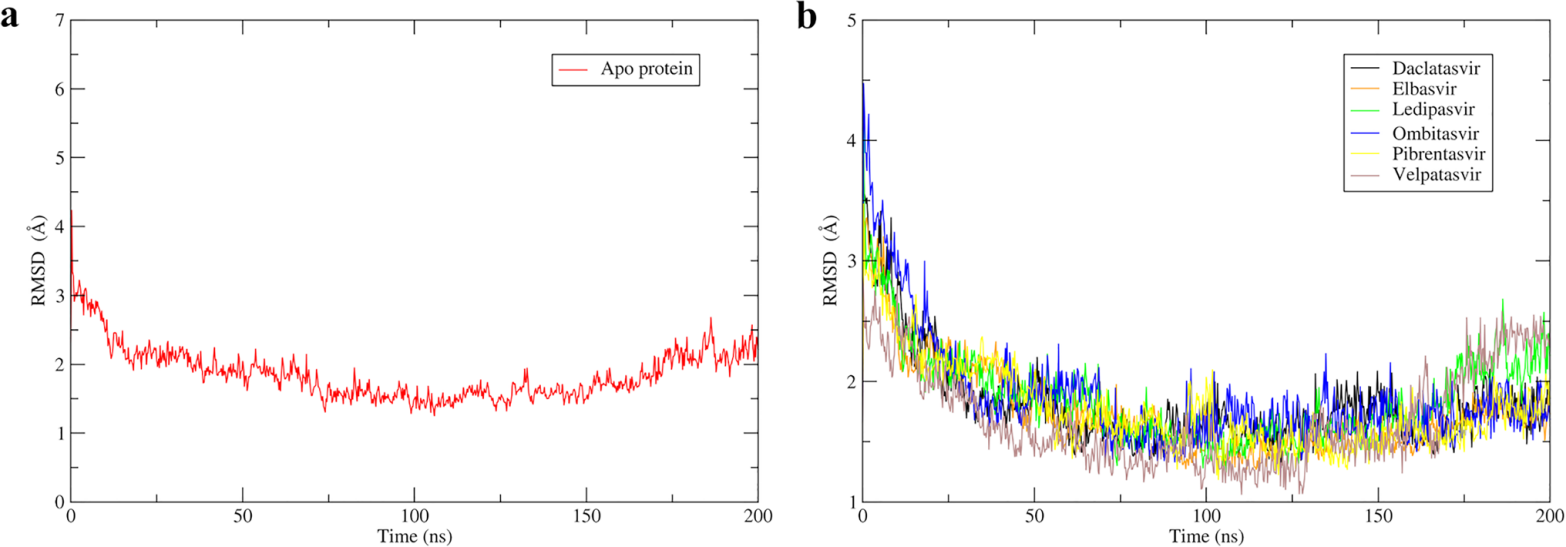
RMSD evolution of apoprotein (**a**) and the protein–ligand complexes (**b**) over the 200-ns MD simulations

Root mean square deviation (RMSD) checks the protein structure’s dynamic behavior and stability based on its deviation from the original structure. RMSD values on the order of 1–3 Å are considered stable, but a number greater than 3 Å suggests that the protein will experience significant conformational changes during a simulation run. The RMSD of the apoprotein backbones is shown in Fig. 10a. The RMSD value fluctuated throughout the first 25 ns of simulation and then became steady; this means that the modeled structure is stable and can be considered for rigorous analysis. The dynamic motion of HCV NS5A GT-4a in response to ligand binding over a 200-ns MD simulation was examined. As shown in Fig. 10b, there are some fluctuations at the beginning. However, all systems reached decent stability at the end of the simulation time; this strongly indicated the conformational stability that the protein had achieved with the ligand molecules.

Furthermore, the root mean square fluctuation (RMSF) of the apoprotein was computed to track the regions of the protein that fluctuated the most during the MD simulations. The N- and C-terminals fluctuated the most. Loops fluctuated more than helices and strands. Apoprotein showed a higher degree of movement at the N- and C-terminals within 3.6 Å (Fig. S21).

The radius of gyration (rGyr) measures the degree of structural compactness of protein before and after ligand binding. The data of the apoprotein and protein–ligand complexes (Fig. S22) demonstrate that the radius of gyration of HCV NS5A GT-4a remained consistent throughout interactions with all compounds. This indicates that the protein does not denature and remains folded. Any denaturation of HCV NS5A GT-4a can terminate the inhibitory pathway, which is not the goal of this study.

Understanding how the ligands interact with the residues inside the binding pocket of HCV NS5A GT-4a can greatly aid in determining the details of the inhibition mechanism. As a result, the amino acid residues interacting with the investigated inhibitors were tracked throughout the MD simulations time, represented in a histogram, and normalized throughout the trajectories (Fig. S23). Furthermore, all interactions that occur for more than 5% of the simulation time are depicted. In the case of daclatasvir, Trp11A, Thr56A, Thr56B, and Cys57B amino acids exhibit H-bonds during 71%, 90%, 24%, and 82% of the simulations time, respectively. In addition, π-π interactions between the phenyl ring and Phe19A and Tyr93A were observed (Fig. S24). The total contacts of daclatasvir with NS5A increased over the MD simulations time, notably after ~ 25 ns, as new π-π stacking interactions developed with Trp11A and Trp19A, and a new H-bond is established with the conserved Cys57B (Fig. S25).

In the case of elbasvir, Tyr93A forms an H-bond for 36% and π-π interaction with the imidazole ring for 56% of the MD simulations time. Further, hydrophobic interactions were observed with the Trp11A and His54B for 39% and 70% of the MD simulations time, respectively. Ombitasvir exhibits H-bonds with Thr56A for 41%, Thr56B for 71%, Ala61B for 83%, and Thr95A for 35%, while Tyr93A and His54B show π-π interaction for 34% of the MD simulations time. Ledipasvir shows H-bonds with Thr56B for 84%, Ala61B for 56%, and π-π interaction with Tyr93A for 43% of the MD simulations time. Velpatasvir shows H-bonds with Thr95A, His54B, and Thr56B through water bridges for 42%, 42%, and 46% of the MD simulations time, respectively. In addition to π-π contacts with Trp9B, His54A, His54B, Tyr93A, and Tyr93B, pibrentasvir shows H-bonds for 72%, 89%, and 42% with Trp11A, Thr56A, and Asn91A, respectively, in addition to π-π interaction with Phe19, Tyr93A, and Tyr93B for 40%, 28%, and 30%, respectively (Figs. S26–S30).

The fundamental interactions indicated in the initial docking models remained unaltered during the MD simulations. In addition, significant bridged water interactions were identified between the ligands and Thr56. The hydrophobic interactions involving ligands and Tyr93 and His54 residues are crucial. As a result, Thr56, Tyr93, His54, and Trp11 have been recognized as key residues for ligand binding.

**Fig. 11:**
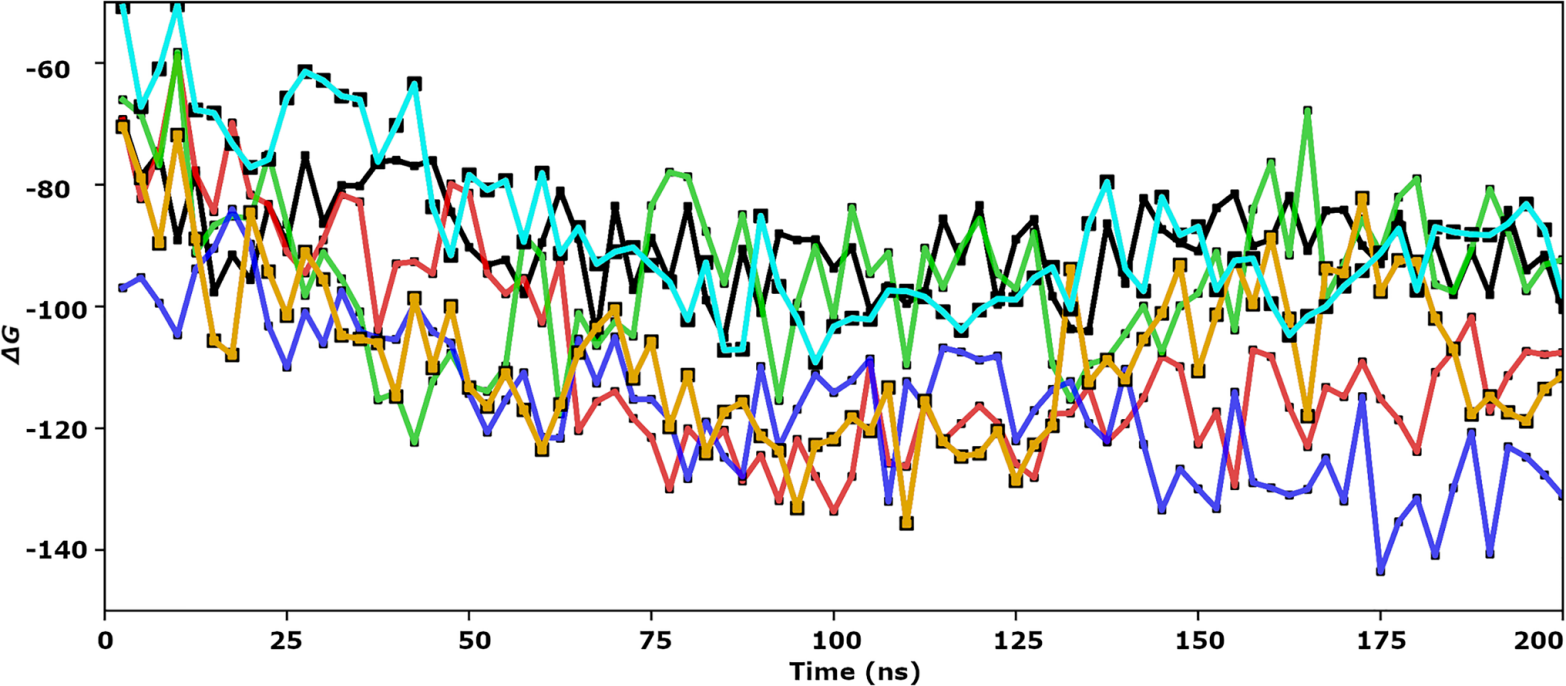
The binding free energy of daclatasvir (orange), elbasvir (black), ledipasvir (red), pibrentasvir (blue), ombitasvir (green), and velpatasvir (cyan) over the 200-ns MD simulations

The MM-GBSA Δ*G* of binding was monitored throughout the MD simulations time (Fig. 11). The average Δ*G* of binding is computed for daclatasvir, elbasvir, ledipasvir, pibrentasvir, ombitasvir, and velpatasvir as − 87.675, − 89.597, − 108.094, − 116.02, − 94.444, and − 107.778 kcal/mol; respectively. The binding energy improved over the MD simulation time, indicating the tight ligand binding.

## Conclusion

Our primary goal was to develop a 3D model of the HCV NS5A GT-4a, considering that the crystal structure of full-length HCV NS5A has not yet been established. Additionally, we use the generated model to explain the binding mode of currently available NS5A inhibitors. As a result, a promising new model for the HCV NS5A GT-4a protein was developed. This model revealed a new unexplored orientation of AH relative to the cytosolic subdomain, with the AH axis approximately perpendicular to the cytosolic subdomain axis (T-shaped). The stability of the generated model was evaluated using 200-ns MD simulations by monitoring the RMSD, RMSF, and rGyr throughout the MD simulations time. Docking predictions through FRED and AutoDock Vina were used to generate and refine the binding modes of marketed NS5A inhibitors. Analysis of the druggable site of NS5A identified a symmetrical cavity. After examining ligand interactions, NS5A inhibitors were found to bind to the NS5A dimer in a symmetrical binding manner. The symmetric properties of daclatasvir and many of its analogs strongly support this binding mode. The binding cavity is located near the point where the dimer attaches to the membrane, and the two N-terminal AH act as ER membrane anchors on both sides of the binding site. The increased potency offered by these bulky groups is thought to result from a more favorable interaction with nearby lipophilic residues in the dimer interface and the AH, supported by a more stable fit to the active site. MD results have confirmed the stability of the studied NS5A inhibitors in the active site. These findings could offer a better understanding of HCV NS5A inhibition mechanism and form a base for the design of potent inhibitors against NS5A, which may confer less resistance and/or a broader spectrum of efficacy against HCV.

## Supplementary information

Below is the link to the electronic supplementary material.

Supplementary file1 (DOCX 22069 KB)

## Data Availability

The datasets generated during and/or analyzed during the current study are available from the corresponding author on reasonable request.

## Code availability

No software code is associated with this article.
