# Supplementary material for: Studies of the symmetric binding mode of daclatasvir and analogs using a new homology model of HCV NS5A GT-4a
Source: J Mol Model. 2022 Dec 29;29(1):25. doi: 10.1007/s00894-022-05420-4 (PMC9800351; doi:10.1007/s00894-022-05420-4)
Supplement: Supplementary file 1 — Supplementary file1 (DOCX 22069 KB) [file 894_2022_5420_MOESM1_ESM.docx]

**Studies of the Symmetric Binding Mode of Daclatasvir and Analogs Using a New Homology Model of HCV NS5A GT-4a**

Kholoud A. Saad^a,*^, Mohammed A. Eldawy^a^, Khaled M. Elokely^a,b,*^

^a^Department of Pharmaceutical Chemistry, Tanta University, Tanta 31527, Egypt

^b^Institute for Computational Molecular Science, and Department of Chemistry, Temple University, Philadelphia, Pennsylvania 19122, United States

*Corresponding Authors Emails:

[kholoud.saad@pharm.tanta.edu.eg](mailto:kholoud.saad@pharm.tanta.edu.eg), [kelokely@temple.edu](mailto:kelokely@temple.edu)


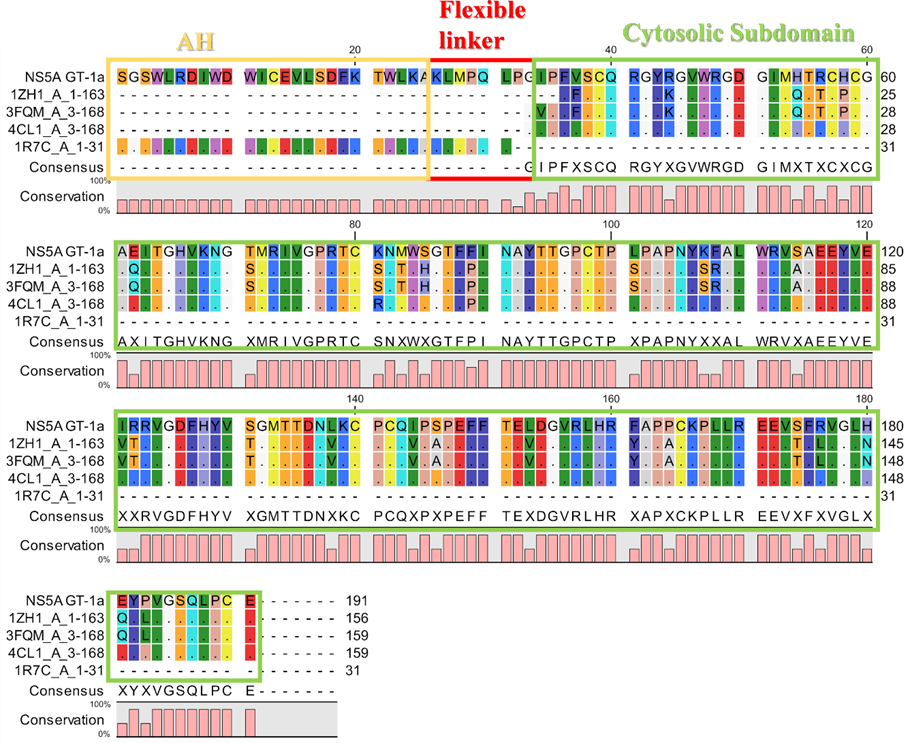


**Fig. S1** The sequence alignment of HCV NS5a GT-1a and the three available crystal structures 1ZH1, 3FQQ, and 4CL1. These sequences are aligned using the T-coffee server [33] and demonstrated using CLC sequence viewer [34]


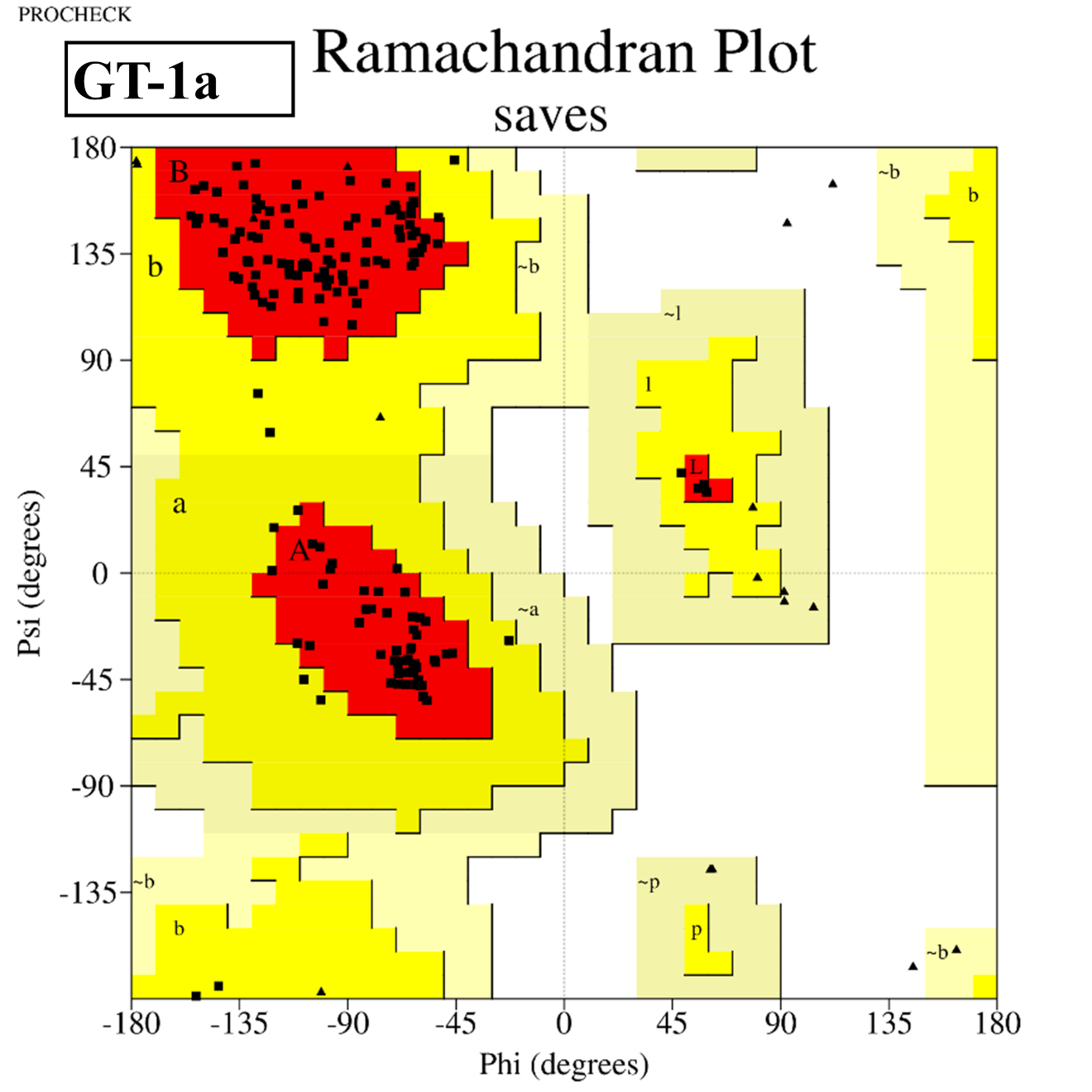


**Fig.S2** Ramachandran plot of the modeled HCV NS5A GT-1a

**
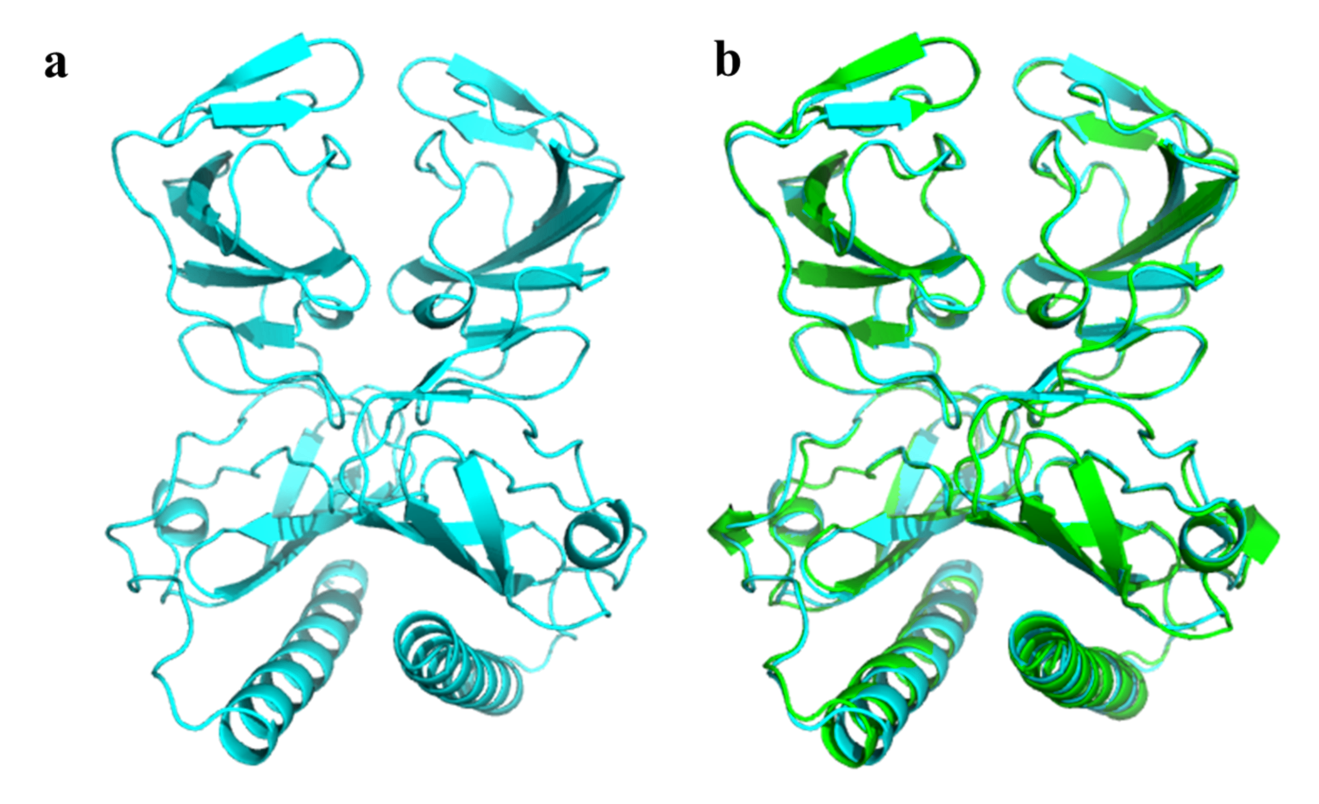
**

**Fig. S3** (a) HCV NS5A GT-4a model using Robetta server [19]. (b) Alignment of GT-1a (green) and GT-4a (cyan)


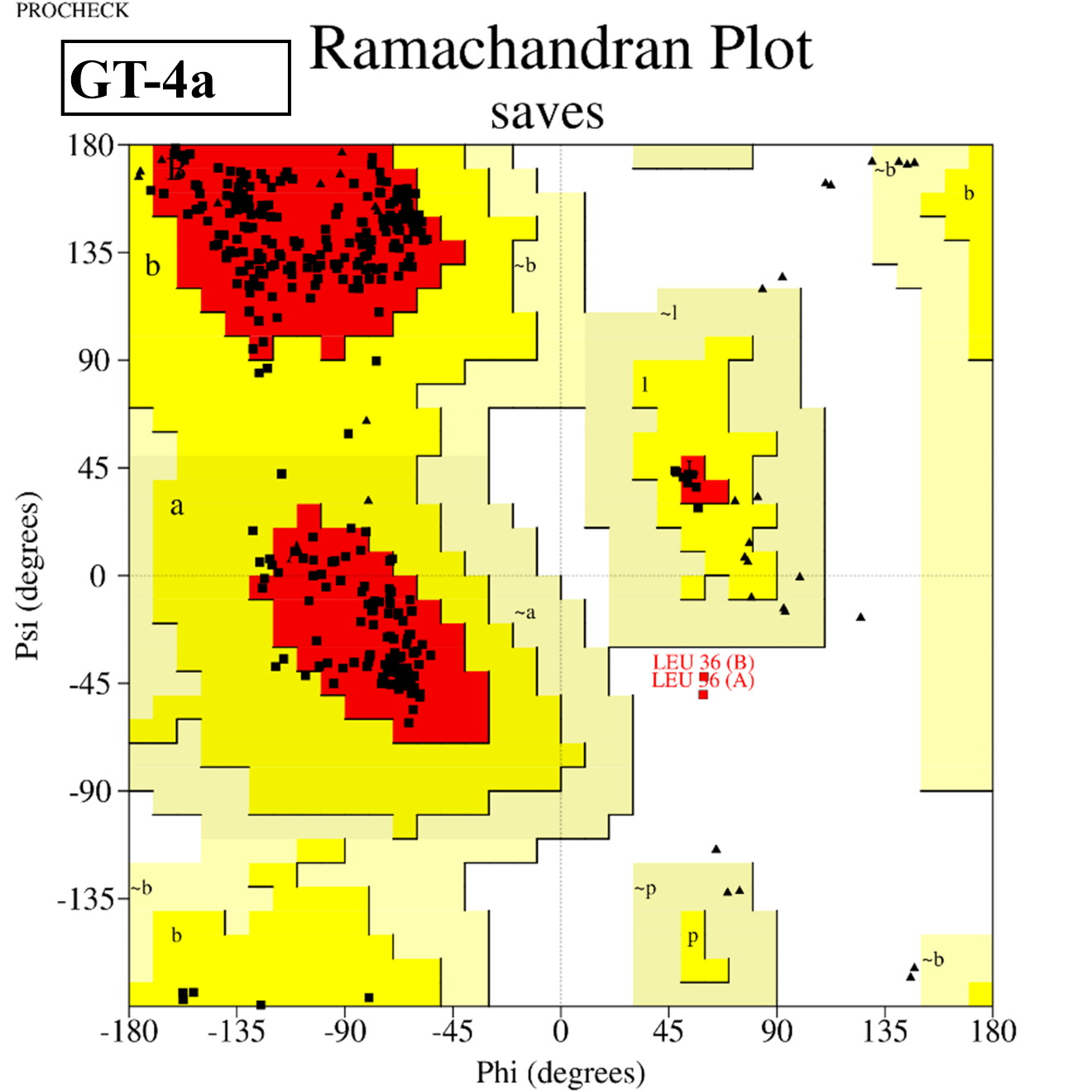


**Fig. S4** Ramachandran plot of the modeled HCV NS5A GT-4a


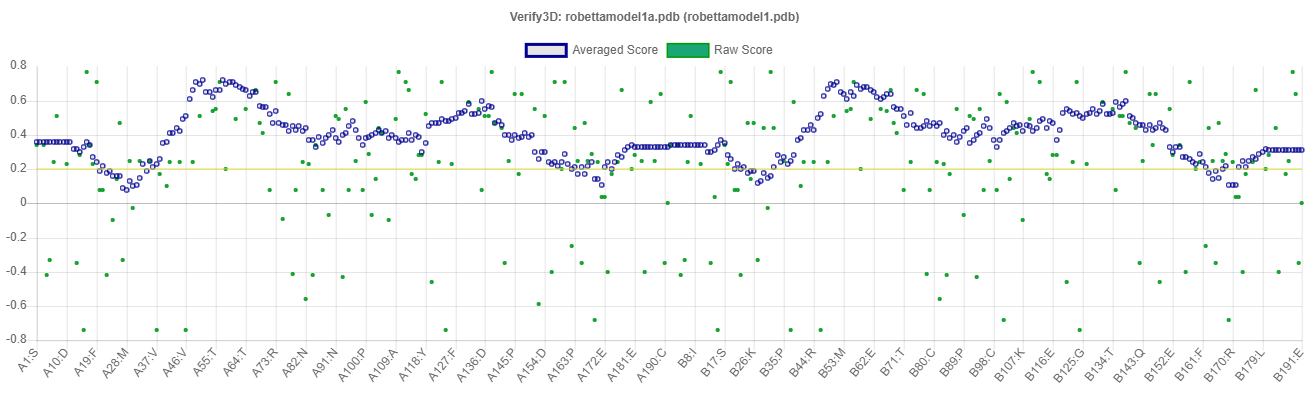


**Fig. S5** Verify3D plot of the modeled HCV NS5A GT-1a


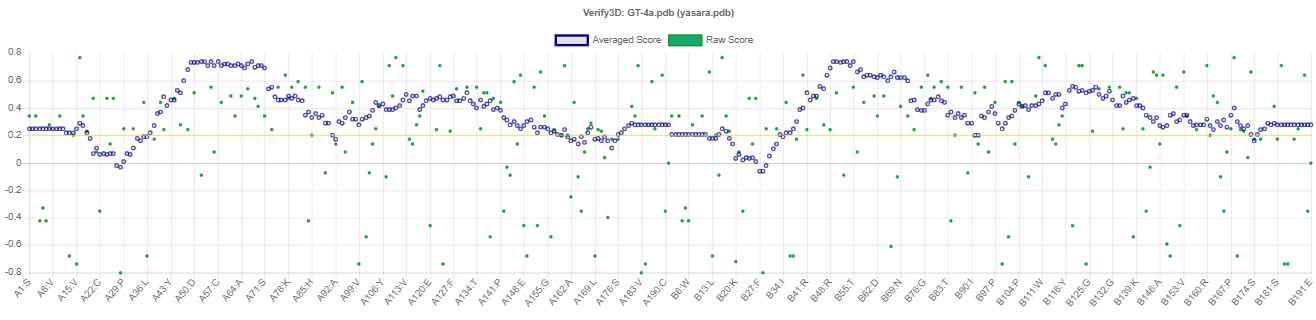


**Fig. S6** Verify3D plot of the modeled HCV NS5A GT-4a


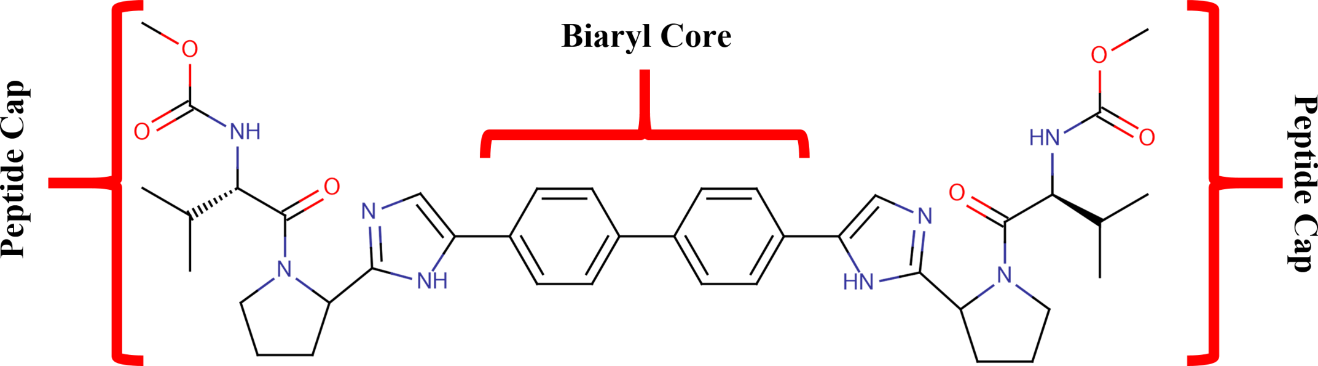


**Fig. S7** Three major segments of daclatasvir


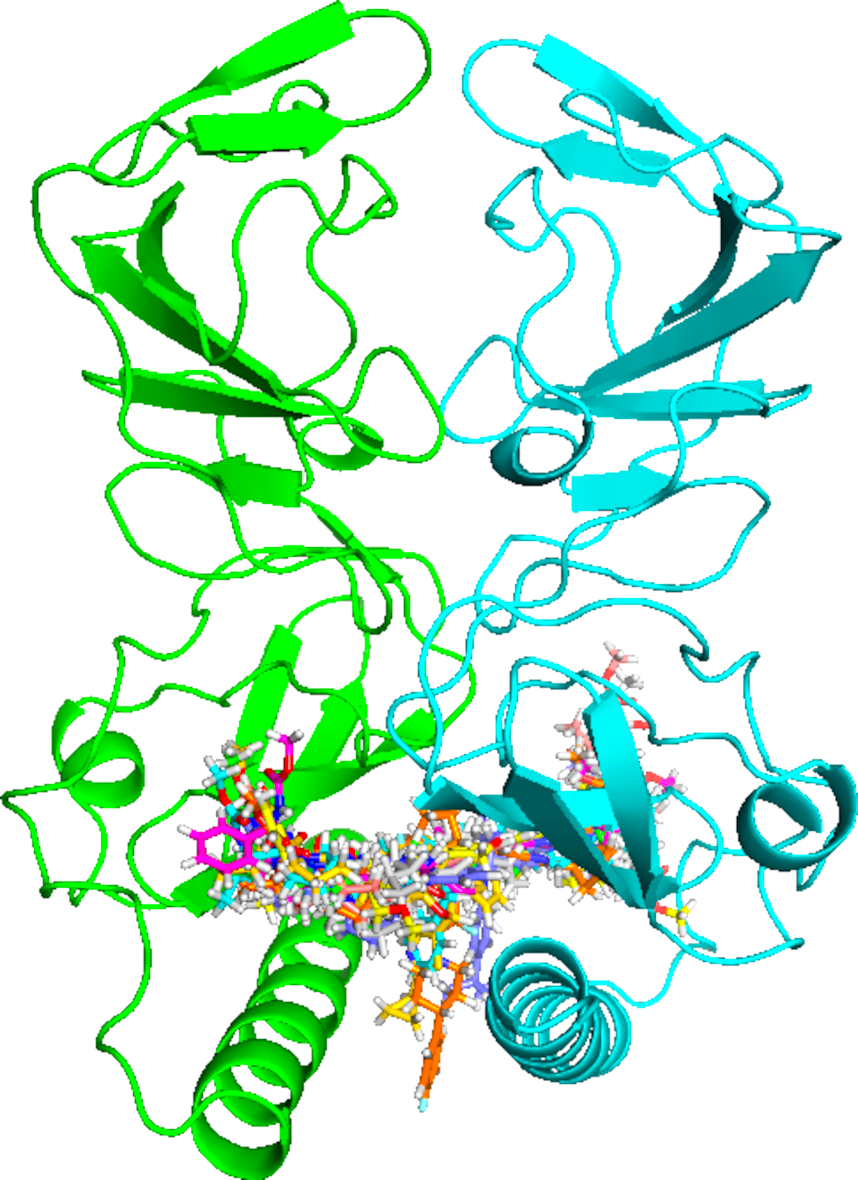


**Fig. S****8** Symmetrical binding mode of daclatasvir and analogs to HCV NS5A GT-4a. The figure was generated using PyMOL


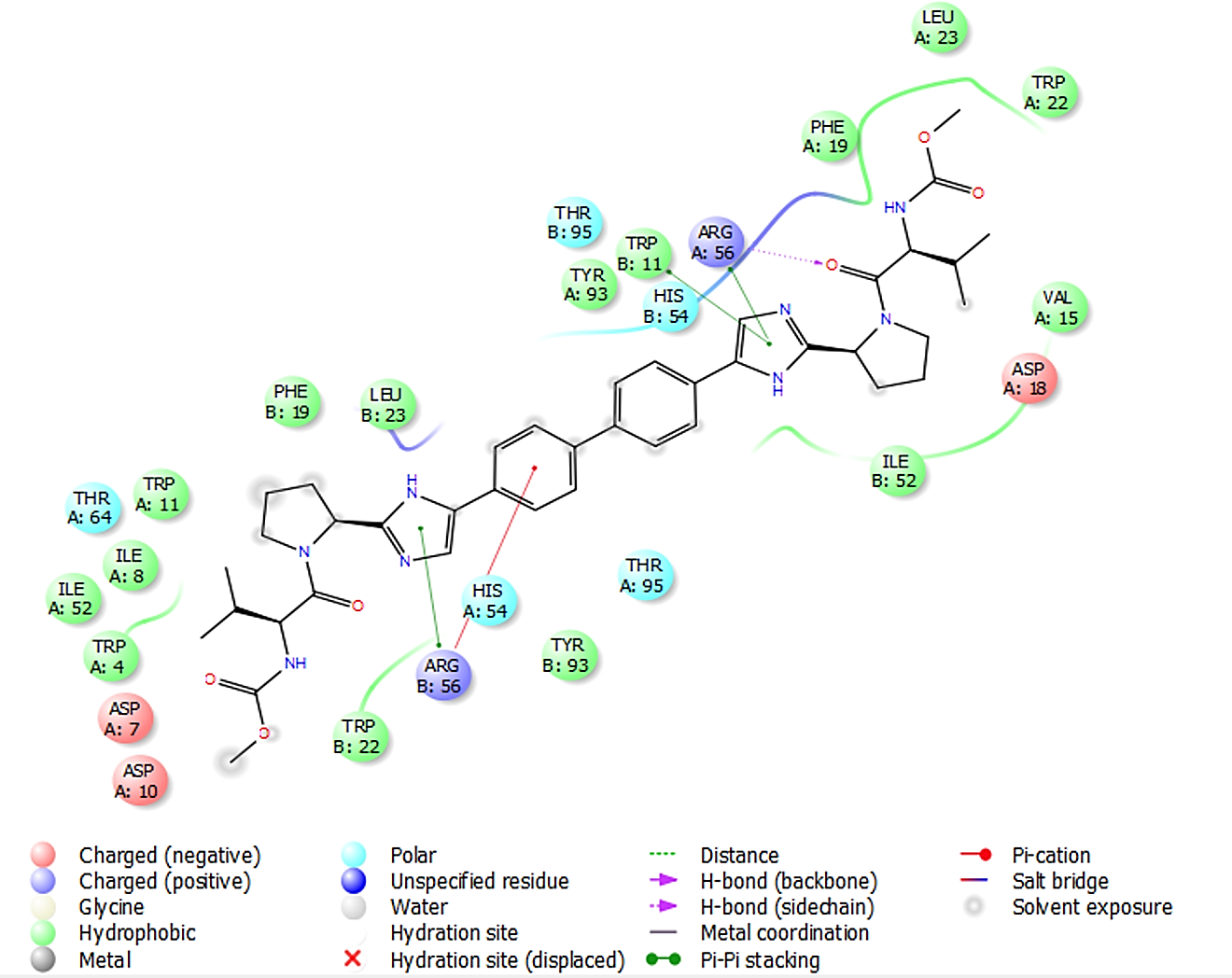


**Fig. S9** The 2D interaction diagram of daclatasvir binding to HCV NS5A GT-1a


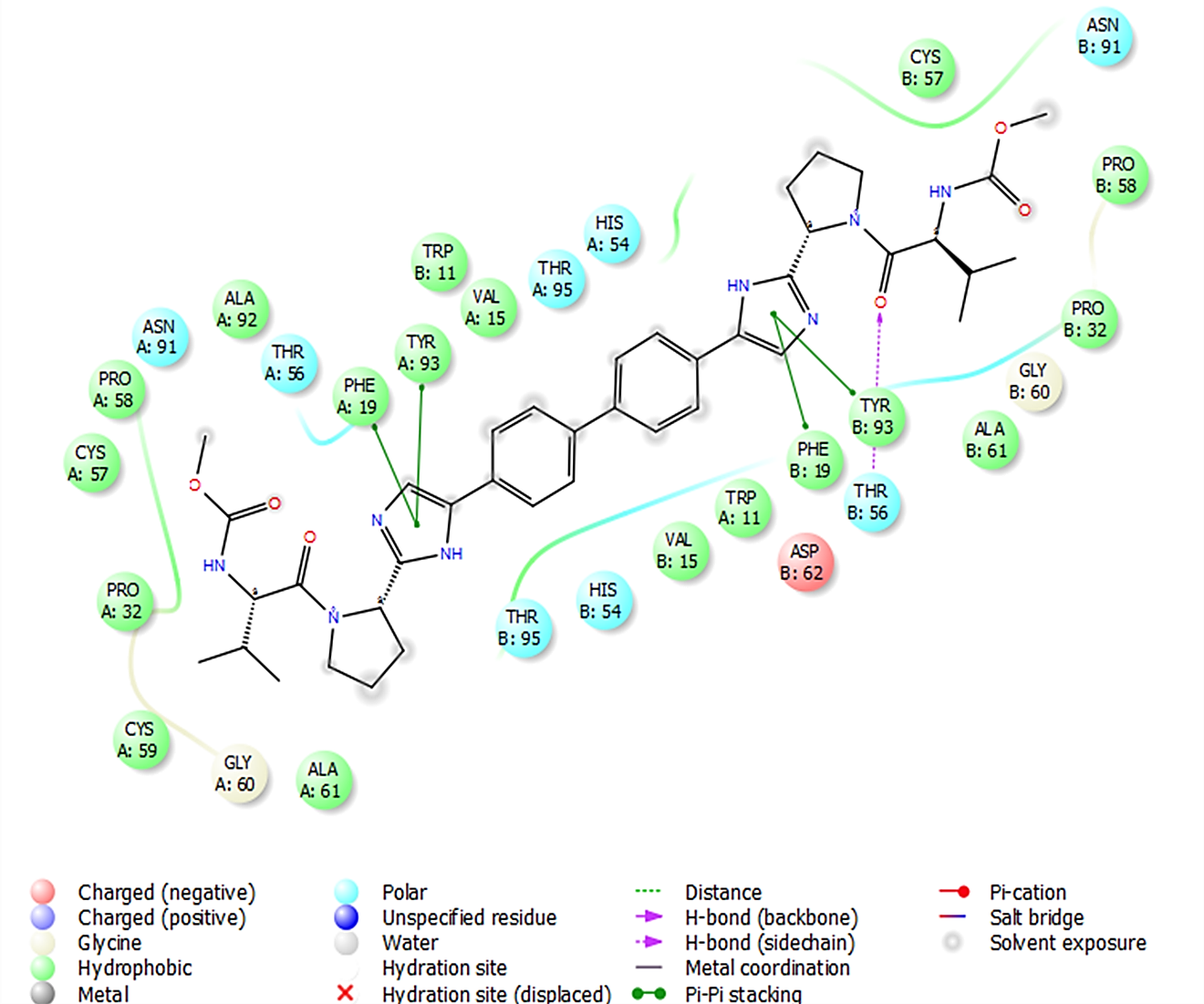


**Fig. S10** The 2D interaction diagram of daclatasvir binding to HCV NS5A GT-4a


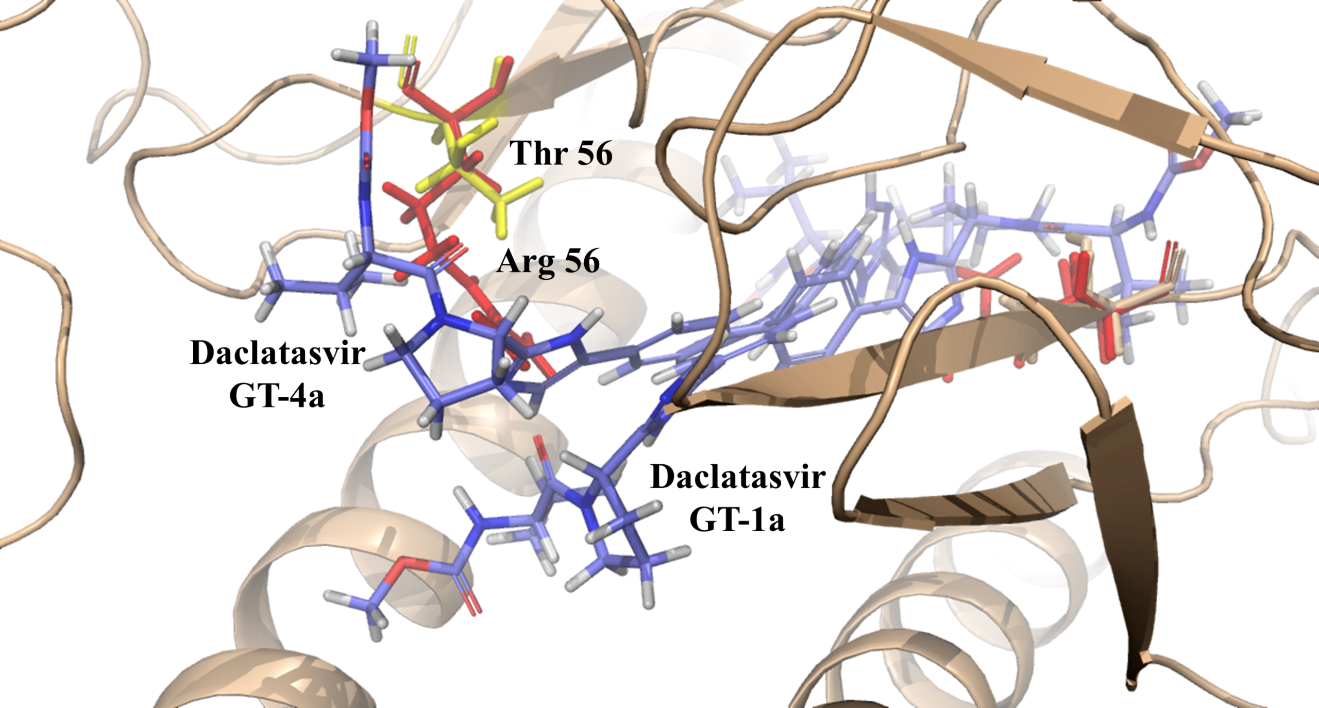


**Fig. S11** Effect of amino acid change in position 56 on daclatasvir binding to HCV NS5A GT-1a and GT-4a. The figure was generated using PyMOL


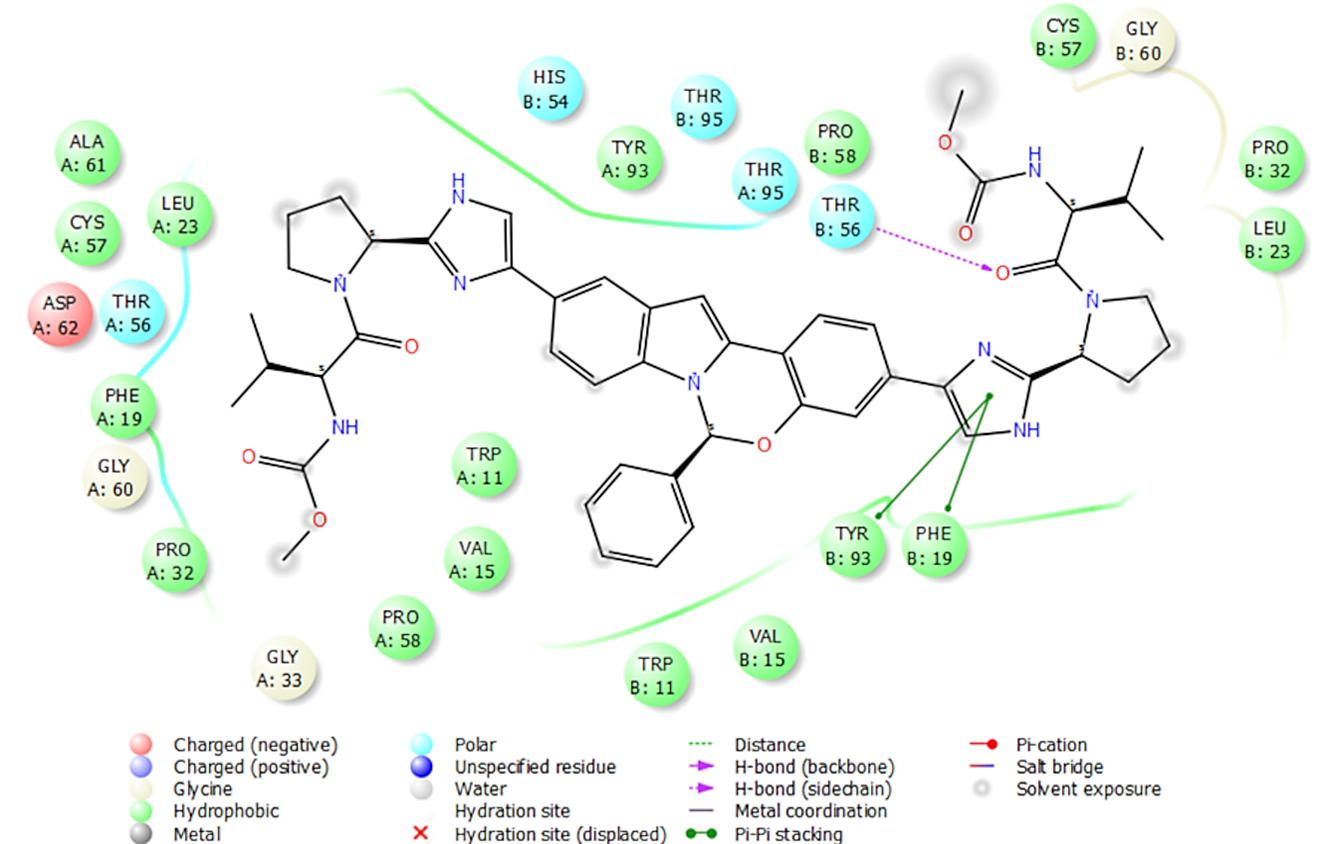


**Fig. S12** The 2D interaction diagram of elbasvir binding to HCV NS5A GT-4a


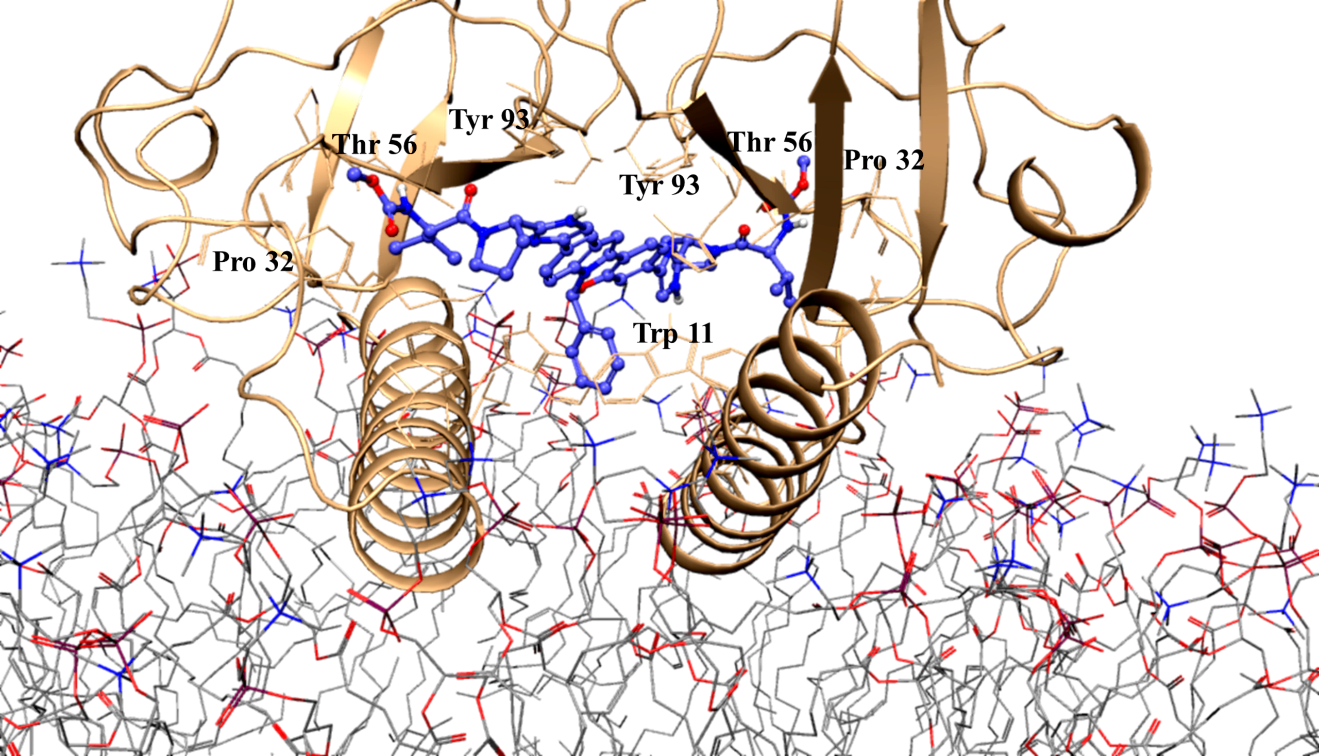


**Fig. S13** NS5A dimer complexed with elbasvir and shows the orientation of the phenyl ring toward the membrane to provide contact with the helix


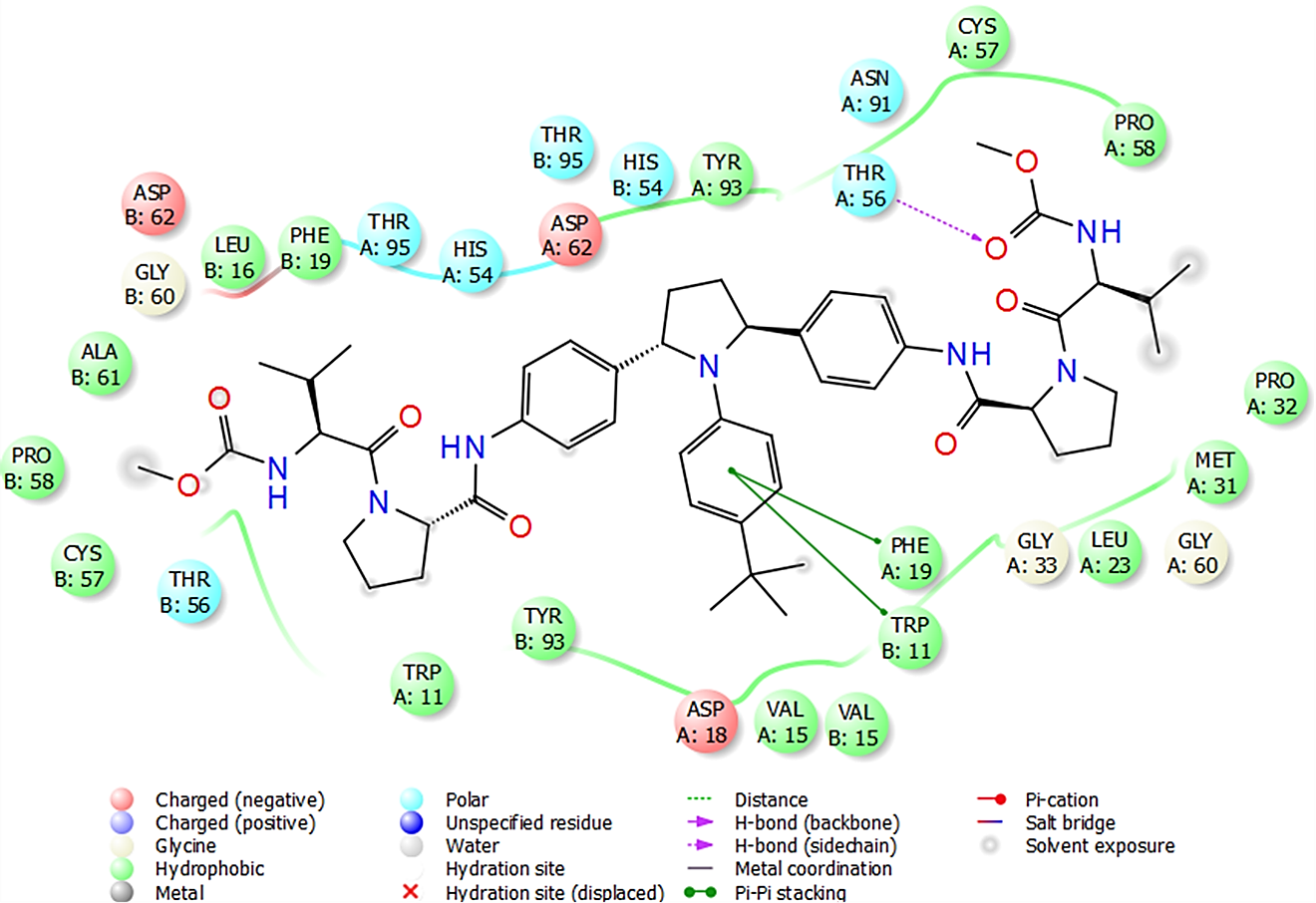


**Fig. S14** The 2D interaction diagram of ombitasvir binding to HCV NS5A GT-4a


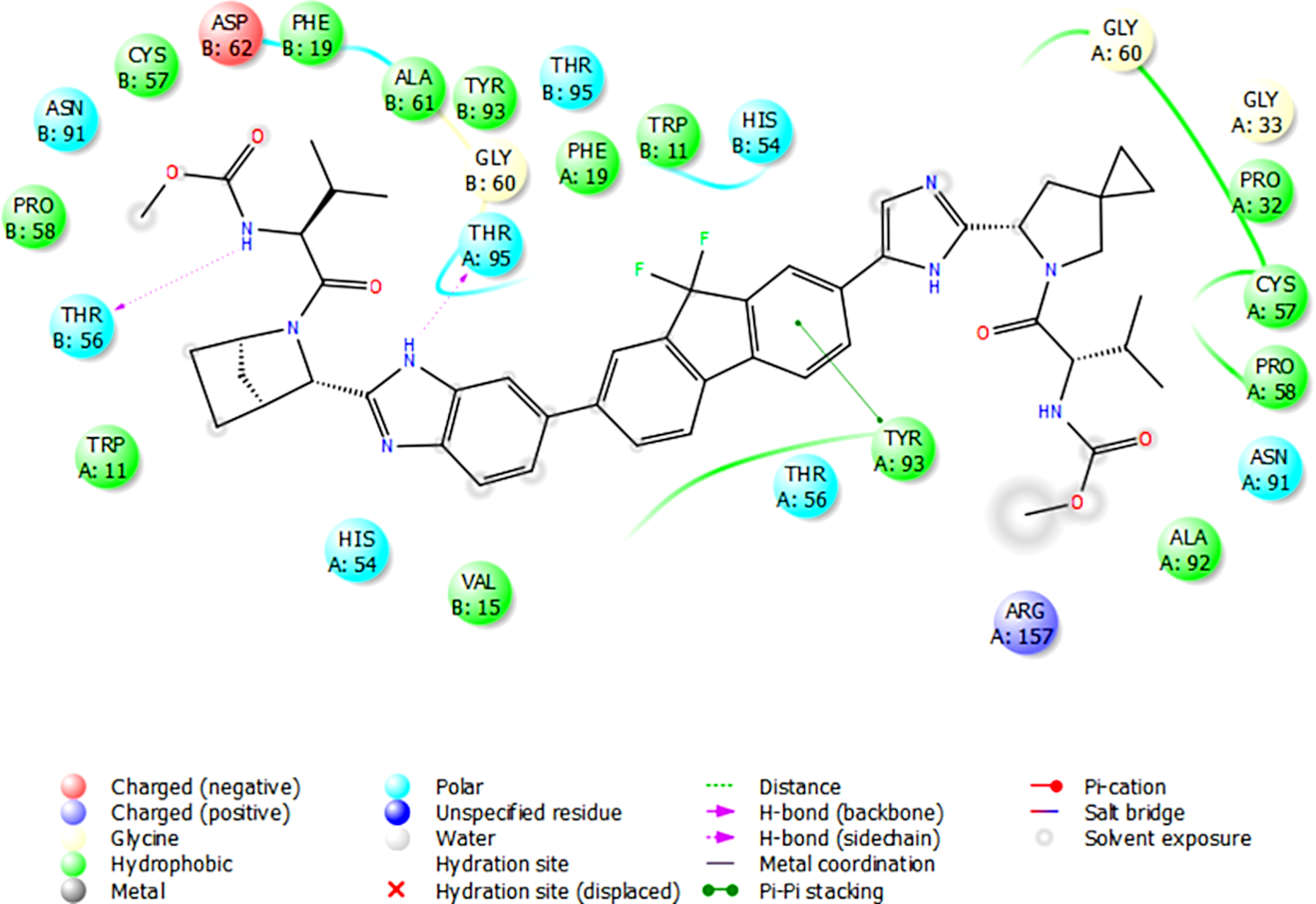


**Fig. S15** The 2D interaction diagram of ledipasvir binding to HCV NS5A GT-4a

**
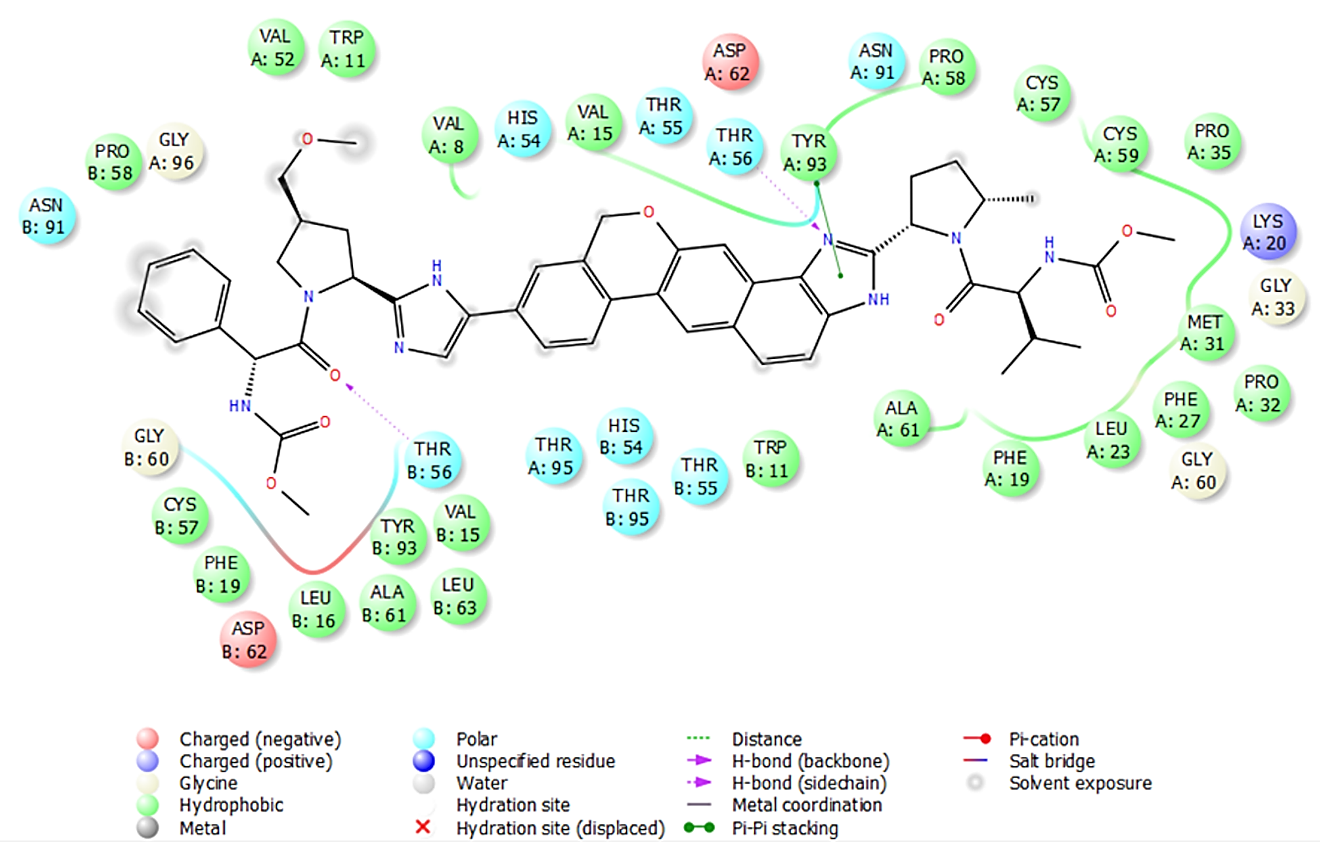
**

**Fig. S16** The 2D interaction diagram of velpatasvir binding to HCV NS5A GT-4a


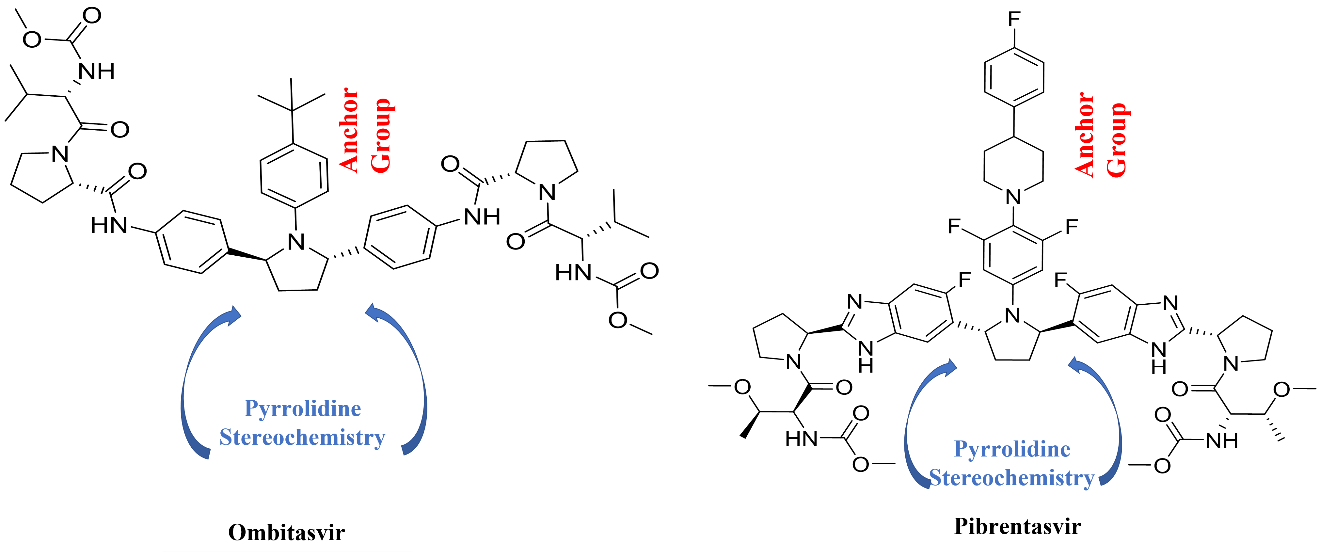


**Fig. S17** Differences between ombitasvir and pibrentasvir structure


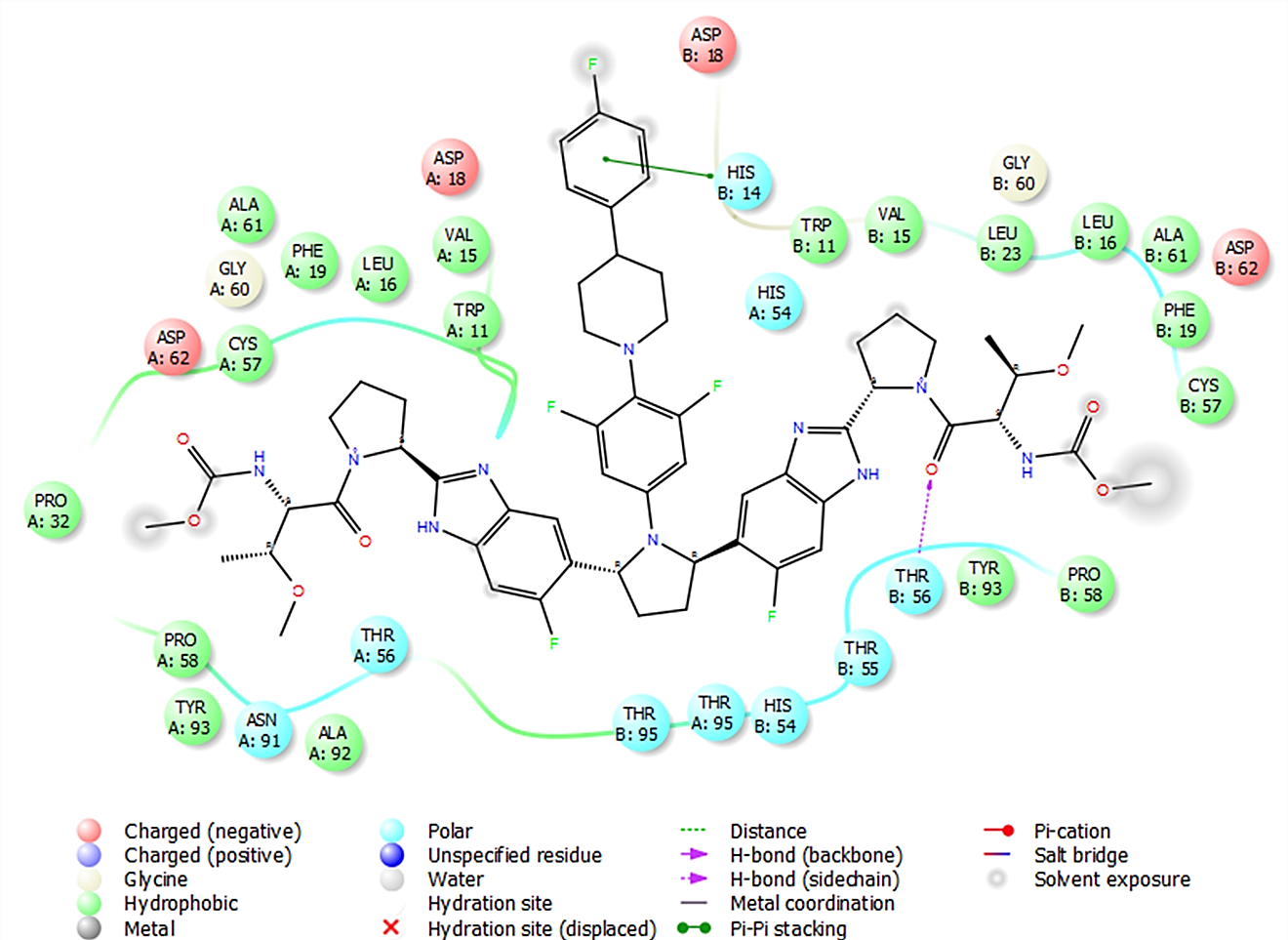


**Fig. S18** 2D interaction diagram of pibrentasvir binding to HCV NS5A GT-4a


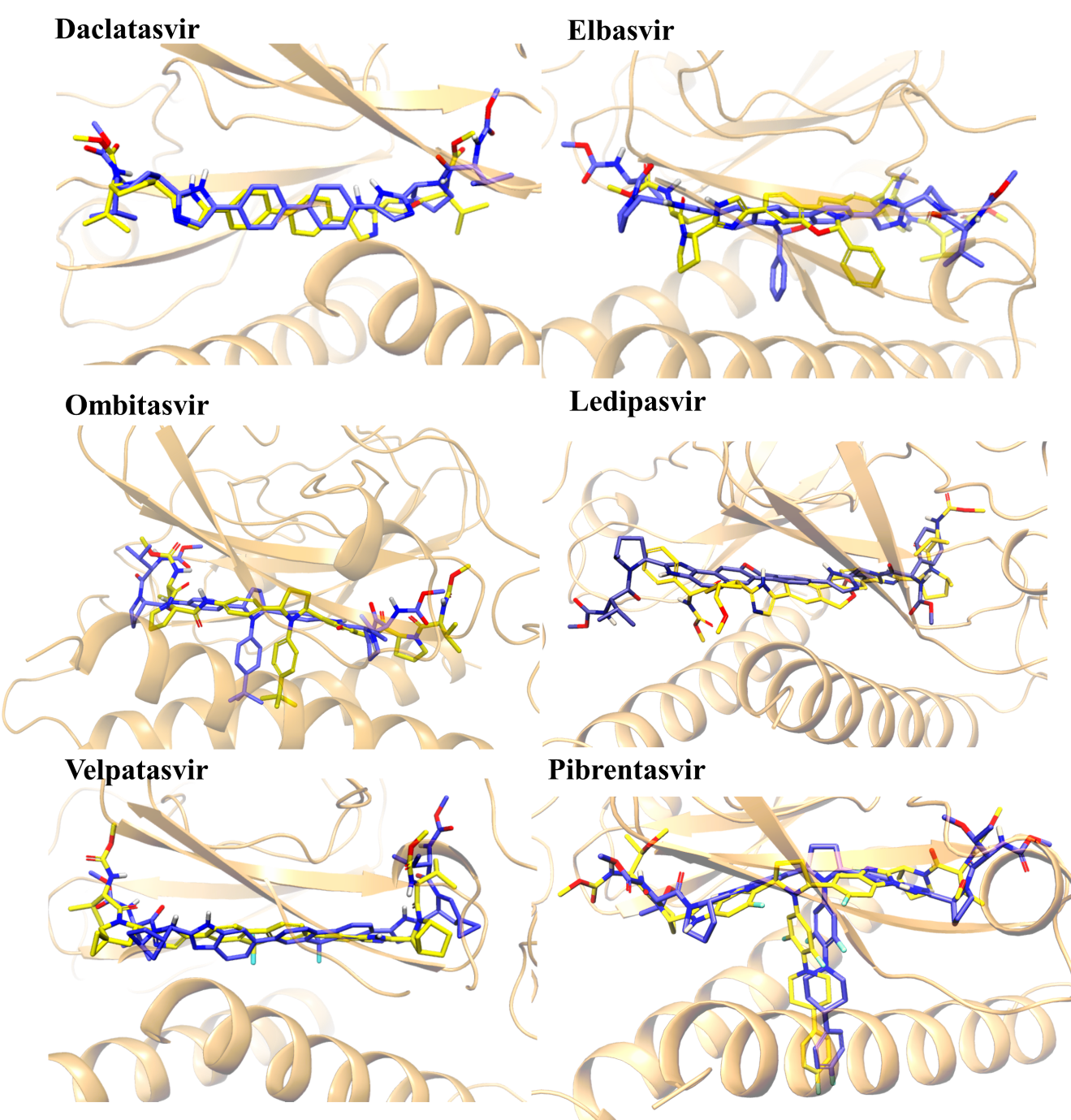


**Fig.** **S19** Comparison between Oedocking (blue) and AutoDock Vina (yellow) results for all studied compounds

**Table. S1** All docking poses of studied compounds and their score using Oedocking and AutoDock Vina

| Compound | Pose | Oedocking docking  (FRED Chemgauss4 score) | Autodock score  (kcal/mol) |
| --- | --- | --- | --- |
| Daclatasvir |  | **-11.9** | **-10.1** |
|  |  | **-11.7** | **-9.9** |
|  |  | **11.6** | **-9.8** |
|  |  | **-11.5** | **-9.6** |
|  |  | **-11.5** | **-9.5** |
|  |  | **-11.4** | **-9.4** |
|  |  | **-11.4** | **-9.3** |
|  |  | **-11.4** | **-9.3** |
|  |  | **-11.3** | **-9.3** |
|  |  | **-11.3** | **-9.2** |
| Elbasvir |  | **-10.4** | **-11.9** |
|  |  | **-9.9** | **-11.8** |
|  |  | **-8.9** | **-11.5** |
|  |  | **-8.9** | **-11.1** |
|  |  | **-8.7** | **-10.8** |
|  |  | **-8.4** | **-10.7** |
|  |  | **-8.4** | **-10.6** |
|  |  | **-8.4** | **-10.5** |
|  |  | **-8.3** | **-10.5** |
|  |  | **-8.3** | **-10.5** |
| Ledipasvir |  | **-11.5** | **-12.0** |
|  |  | **-11.3** | **-11.9** |
|  |  | **-10.8** | **-11.7** |
|  |  | **-10.8** | **-11.7** |
|  |  | **-10.8** | **-11.5** |
|  |  | **-10.7** | **-11.5** |
|  |  | **-10.6** | **-11.3** |
|  |  | **-10.6** | **-11.3** |
|  |  | **-10.5** | **-11.3** |
|  |  | **-9.8** | **-11.2** |
| Ombitasvir |  | **-8.1** | **-10.5** |
|  |  | **-7.9** | **-10.4** |
|  |  | **-7.4** | **-10.2** |
|  |  | **-7.3** | **-9.7** |
|  |  | **-7.2** | **-9.6** |
|  |  | **-7.1** | **-9.5** |
|  |  | **-7.1** | **-9.4** |
|  |  | **-7.1** | **-9.4** |
|  |  | **-7.1** | **-9.3** |
|  |  | **-6.8** | **-9.2** |
| velpatasvir |  | **-9.48** | **-11.3** |
|  |  | **-9.28** | **-11.0** |
|  |  | **-8.5** | **-10.9** |
|  |  | **-8.5** | **-10.5** |
|  |  | **-8.3** | **-10.4** |
|  |  | **-8.3** | **-10.4** |
|  |  | **-8.14** | **-10.4** |
|  |  | **-8.09** | **-10.3** |
|  |  | **-8.09** | **-10.2** |
|  |  | **-8.03** | **-10.1** |
| Pibrentasvir |  | **-6.02** | **-9.8** |
|  |  | **-6.02** | **-9.6** |
|  |  | **-4.3** | **-9.5** |
|  |  | **-4.3** | **-9.5** |
|  |  | **-3.2** | **-9.4** |
|  |  | **-3.2** | **-9.4** |
|  |  | **-3.2** | **-9.3** |
|  |  | **-2.9** | **-9.2** |
|  |  | **-2.9** | **-9.1** |
|  |  | **-2.9** | **-8.9** |


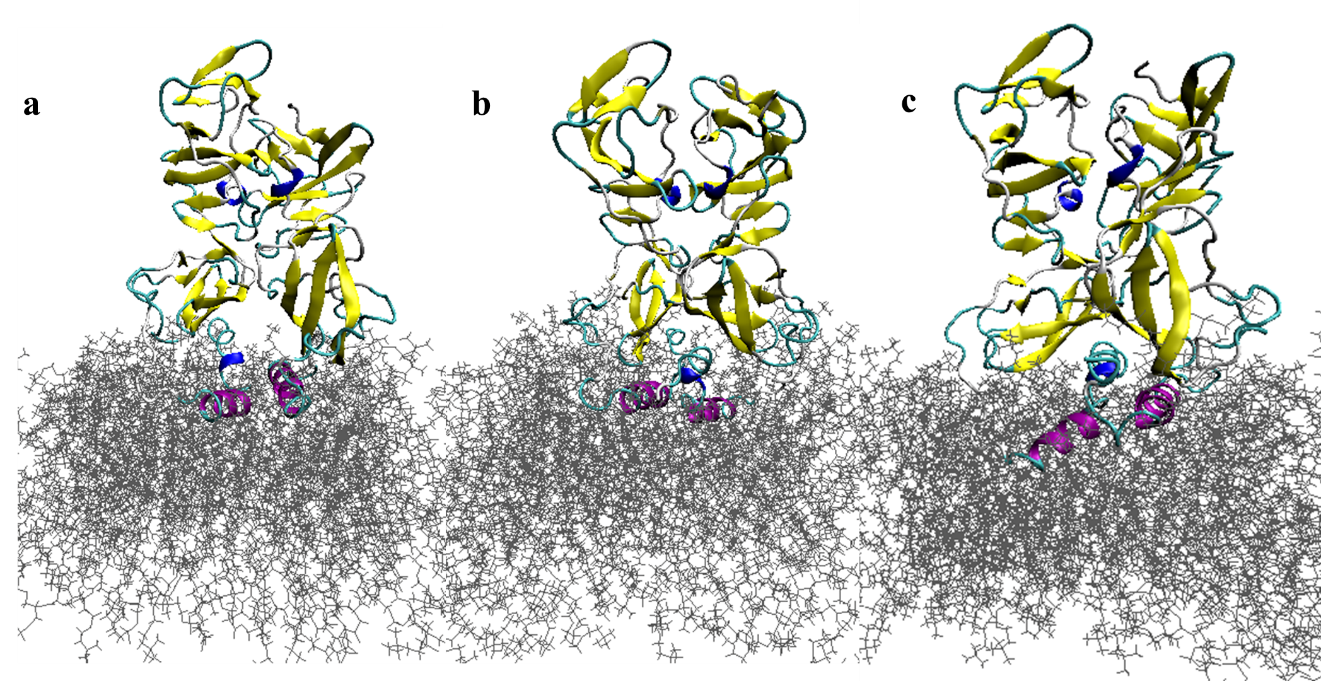


**Fig. S20** Position of the AH relative to the membrane during 200 ns simulation (a) at the beginning (b) after 125 ns (c) at the end of the simulation


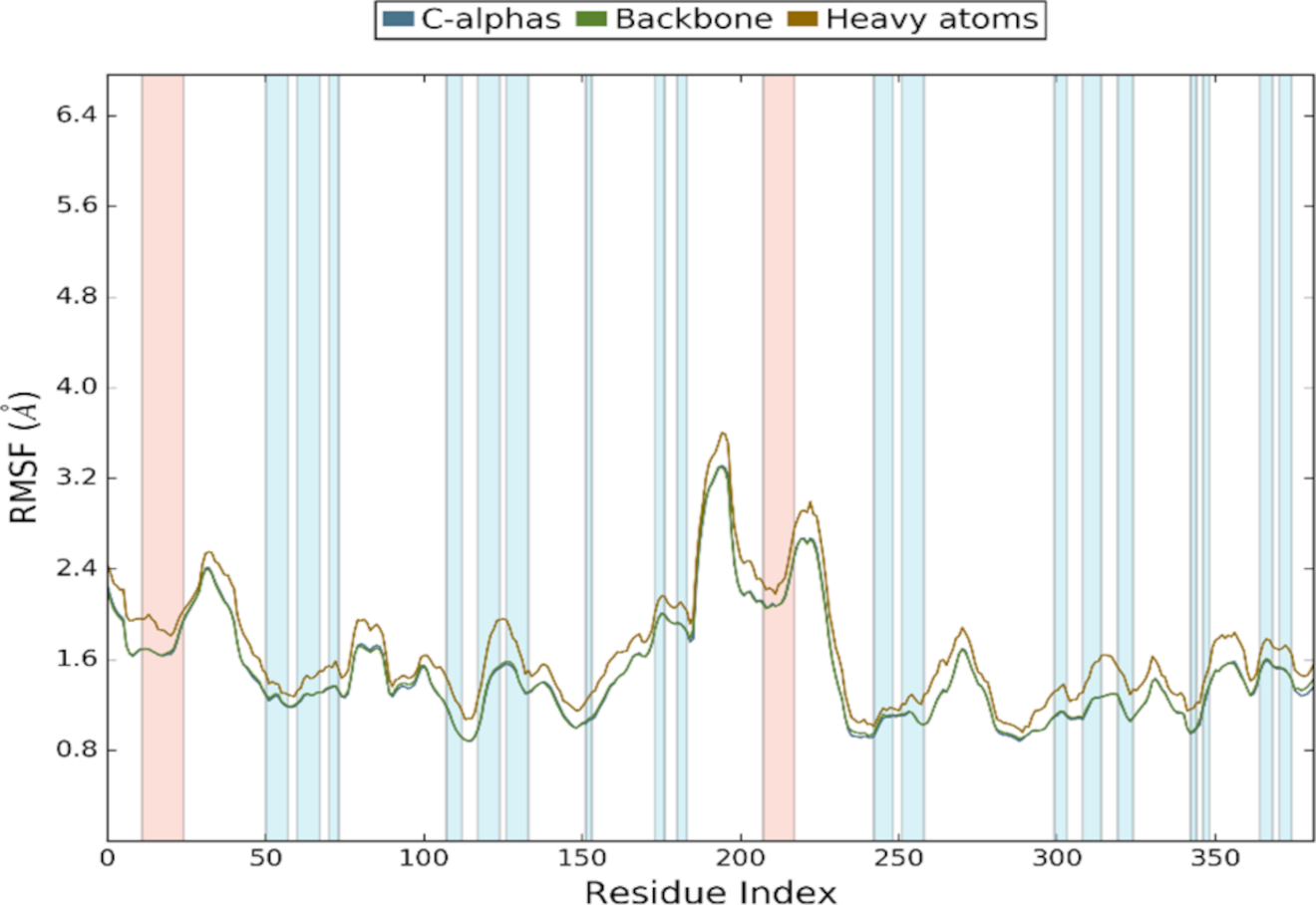


**Fig. S21** RMSF plot for the modeled HCV NS5A protein GT-4a. The alpha-helical and beta-strand regions are highlighted in red and blue backgrounds, respectively


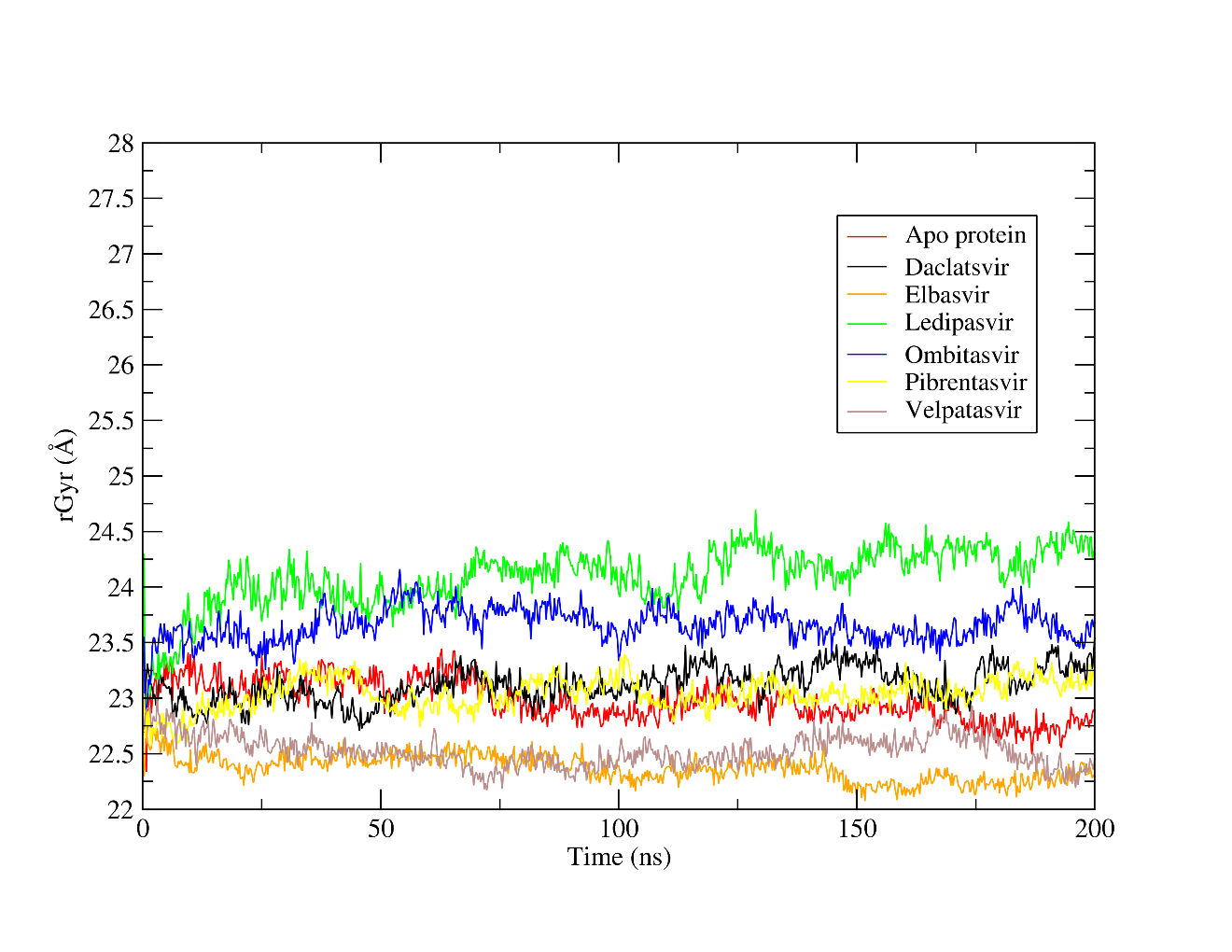


**Fig. S22** Radius of gyration (rGyr) for apo protein and protein-ligand complexes during 200 ns simulation time


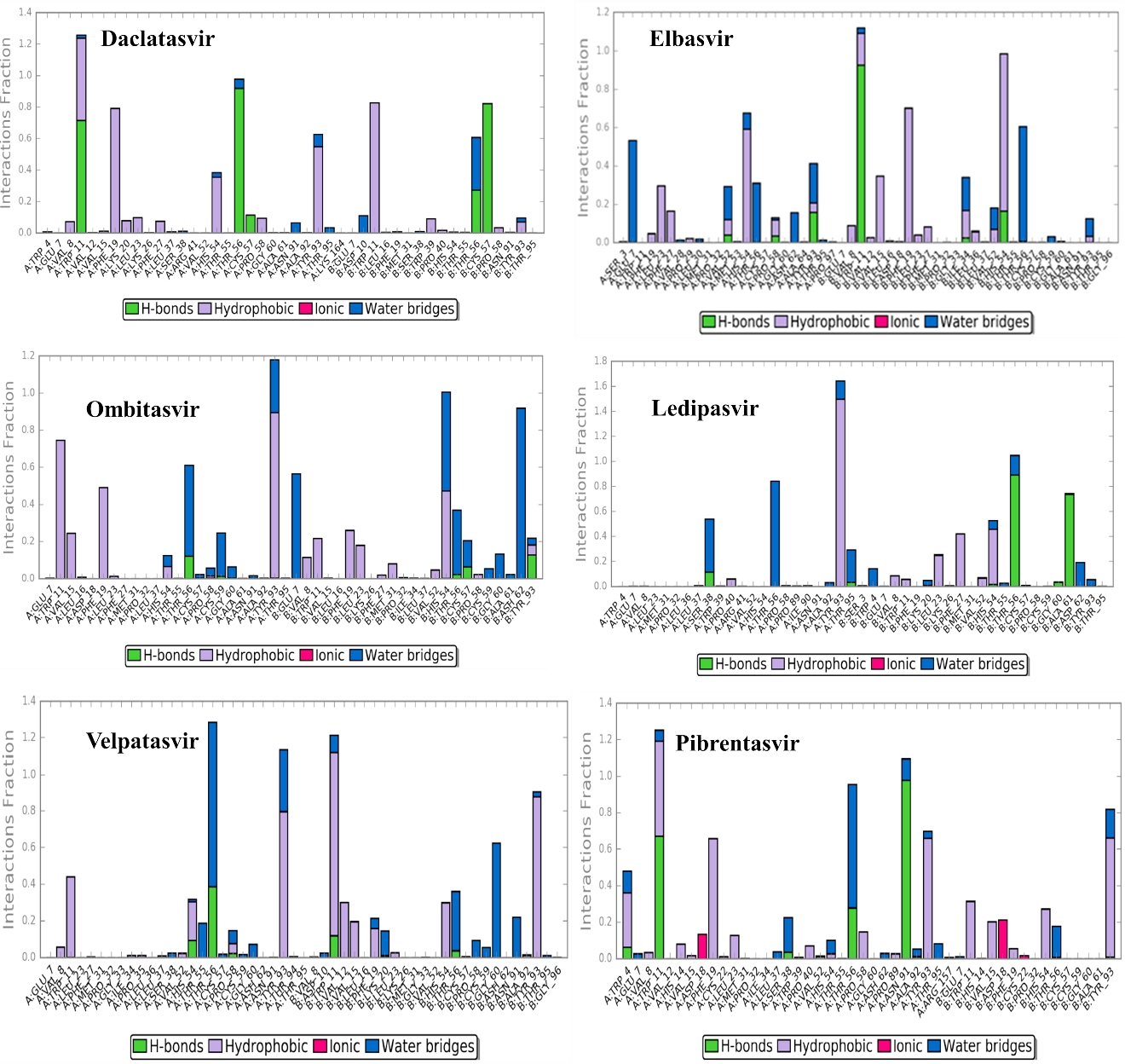


**Fig. S23** Protein-ligand contacts of HCV NS5A GT-4a with respective compounds daclatasvir, elbasvir, ombitasvir, ledipasvir, velpatasvir, and pibrentasvir


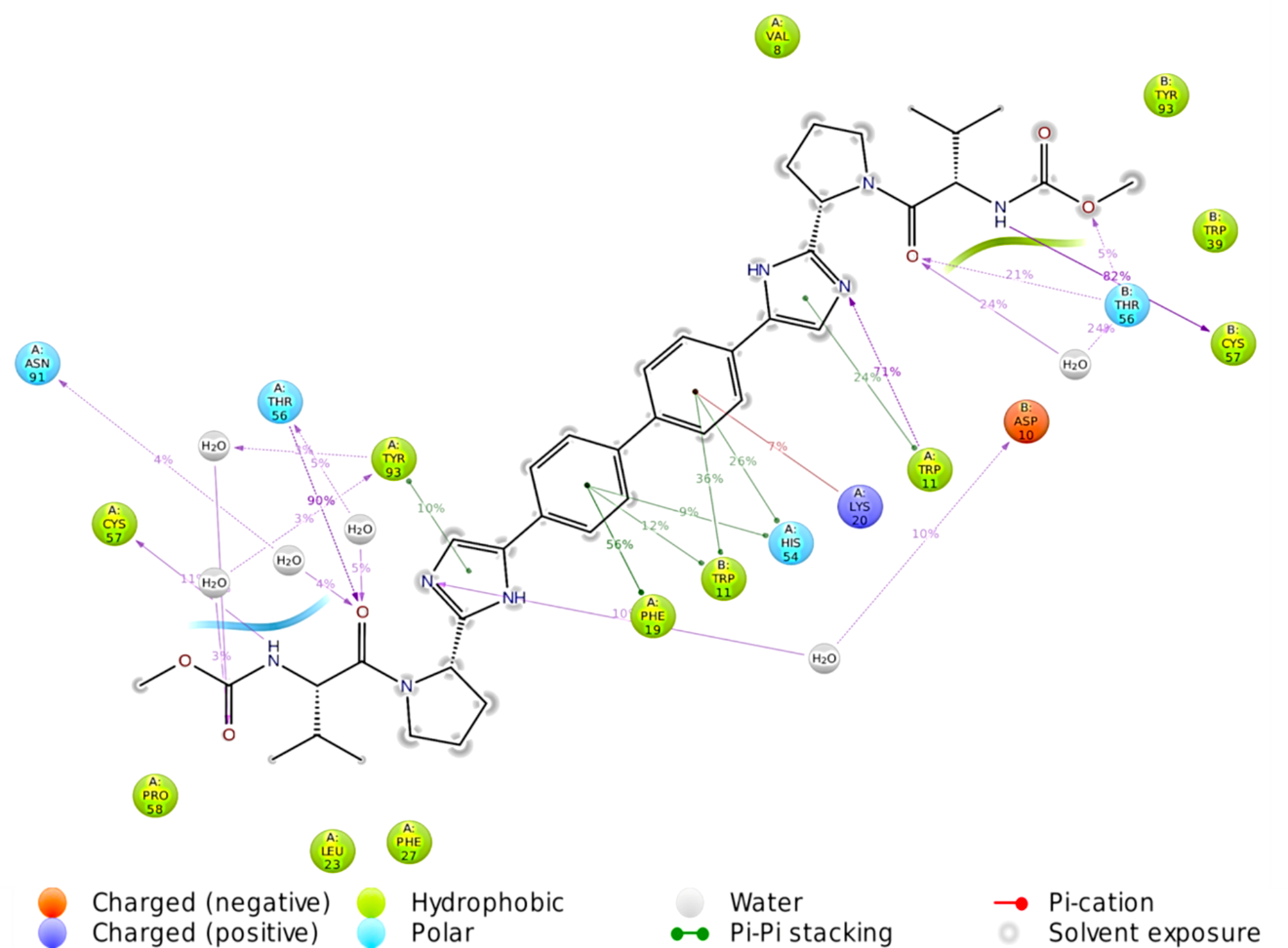


**Fig. S24** Protein-ligand contacts of HCV NS5A GT-4a with daclatasvir


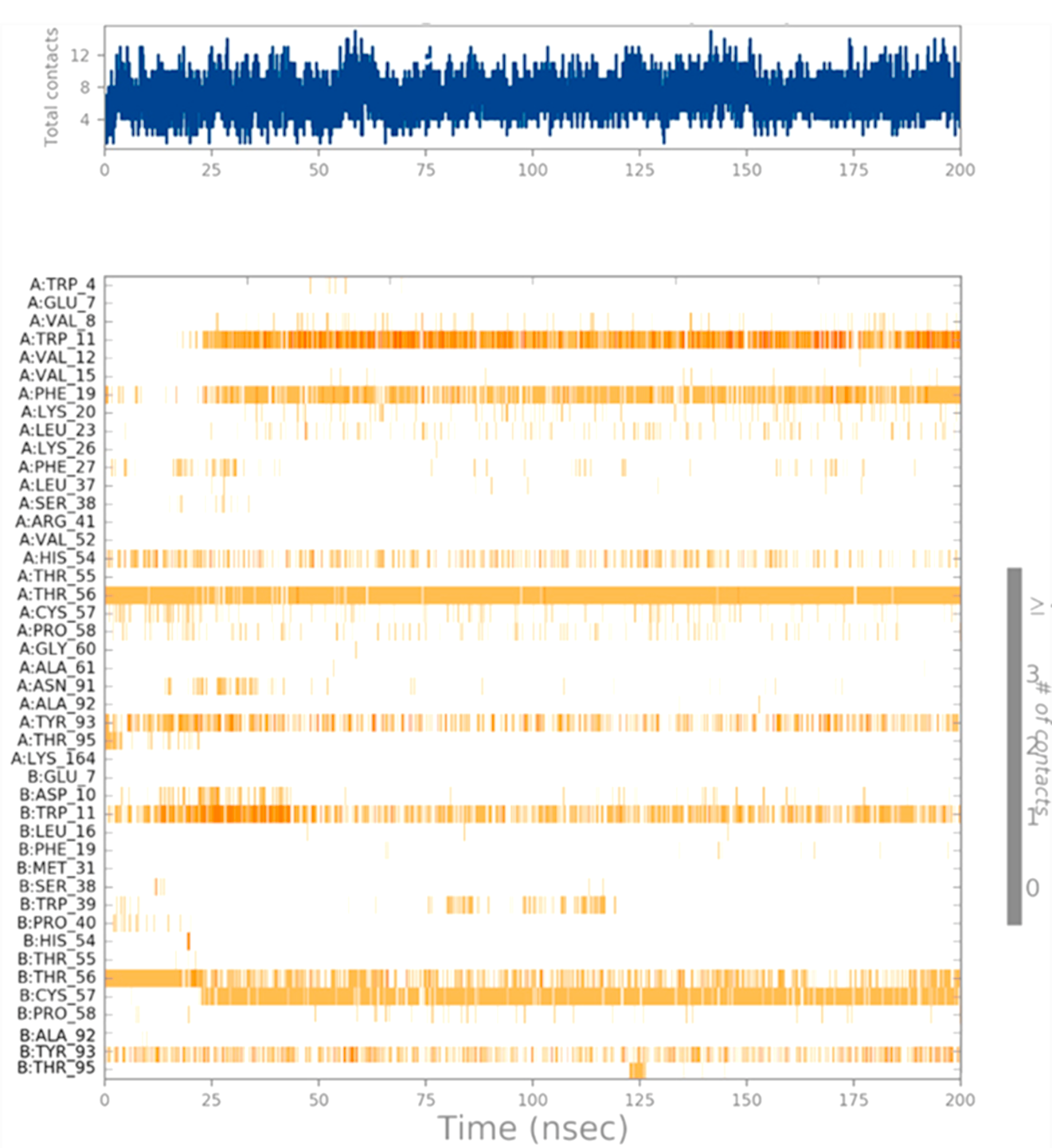


**Fig. S25** Timeline representation of daclatasvir—Receptor interactions. The top section depicts the total number of contacts made by the protein with the ligand as the simulation progresses. In each trajectory, the bottom section depicts residues interacting with the ligand. According to the scale to the right of the plot, several residues make several contacts with the ligand, which is represented by a darker shade of orange

**Fig. S26** Protein-ligand contacts of HCV NS5A GT-4a with elbasvir


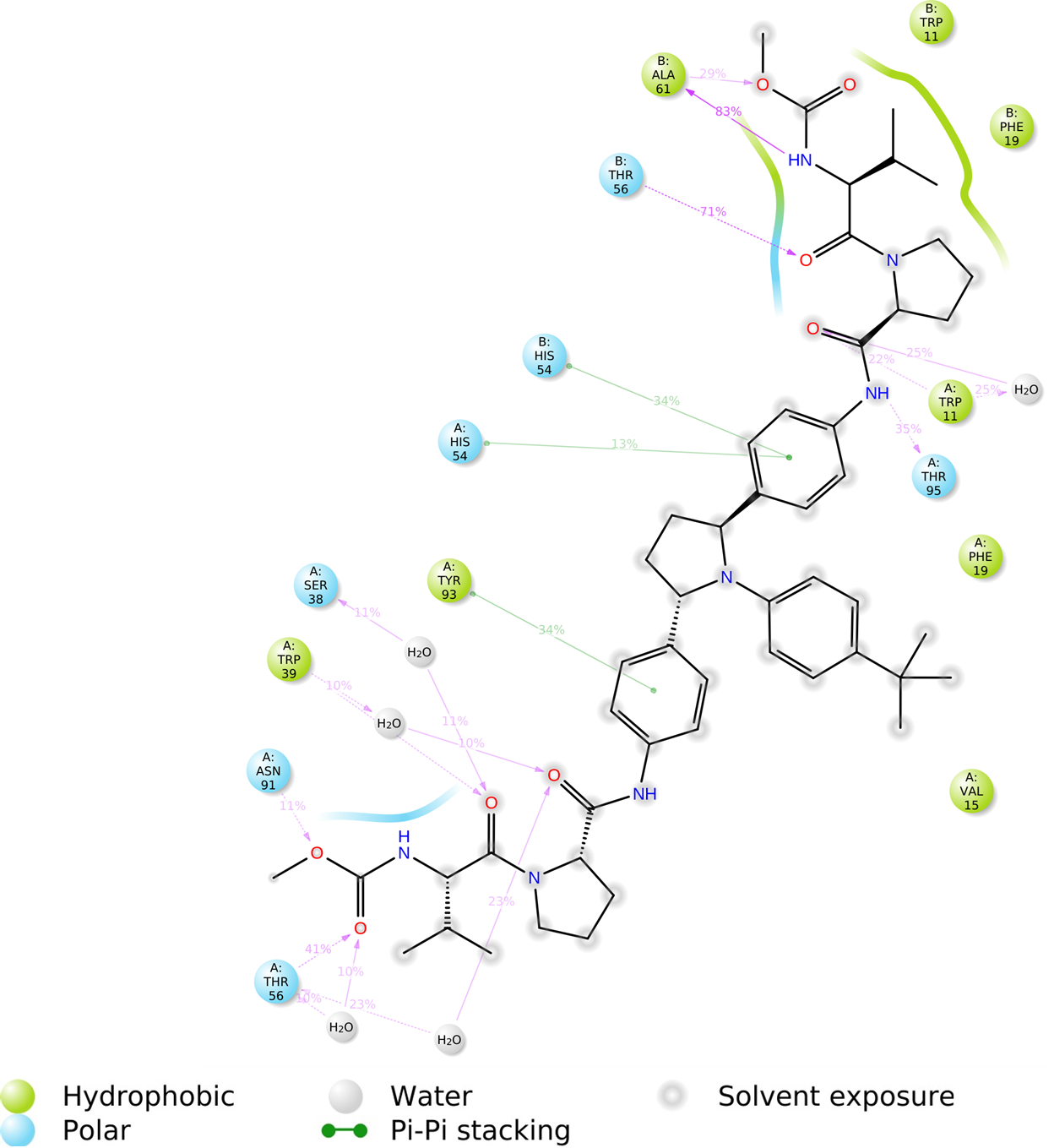


**Fig. S27** Protein-ligand contacts of HCV NS5A GT-4a with ombitasvir


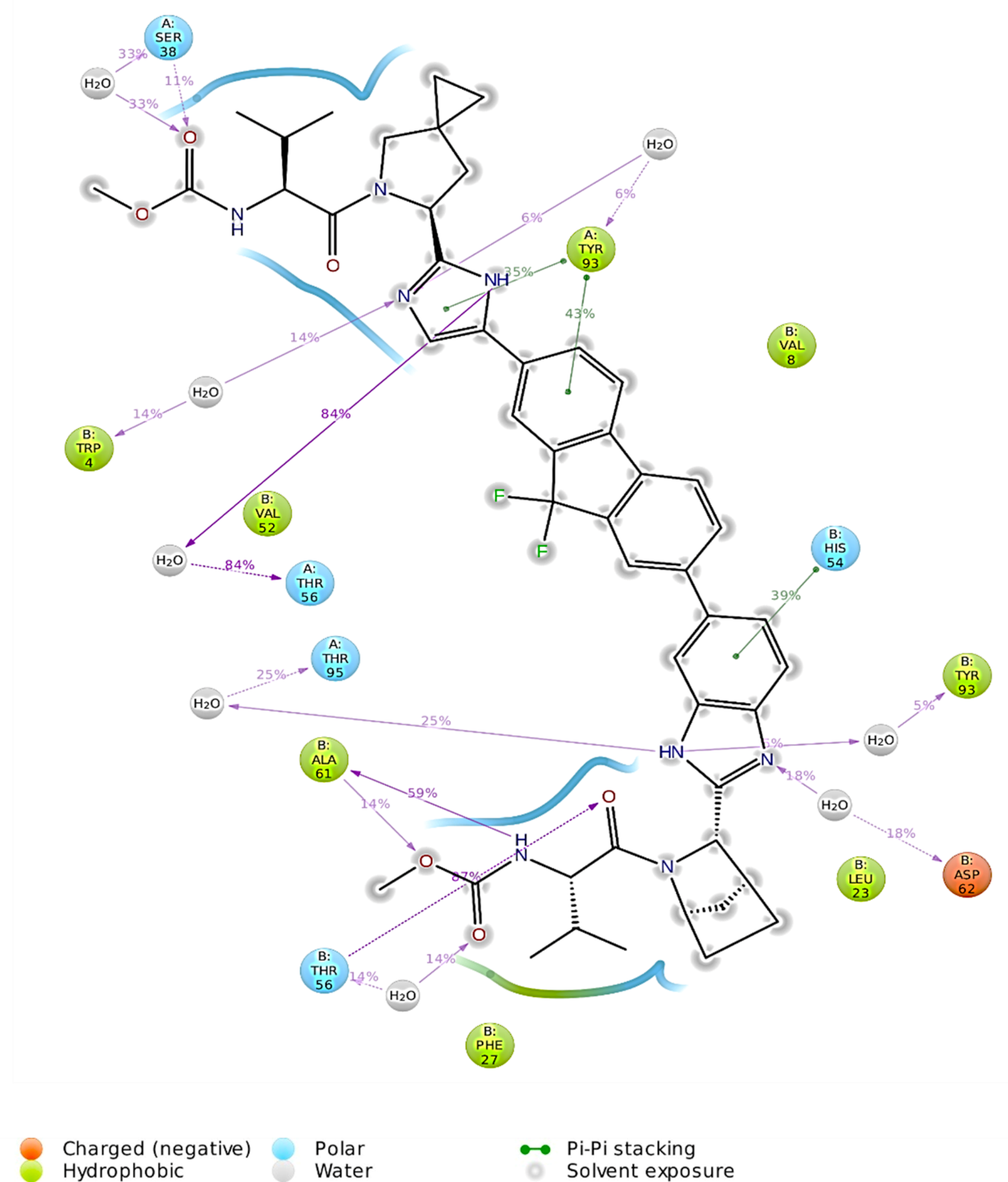


**Fig. S28** Protein-ligand contacts of HCV NS5A GT-4a with ledipasvir


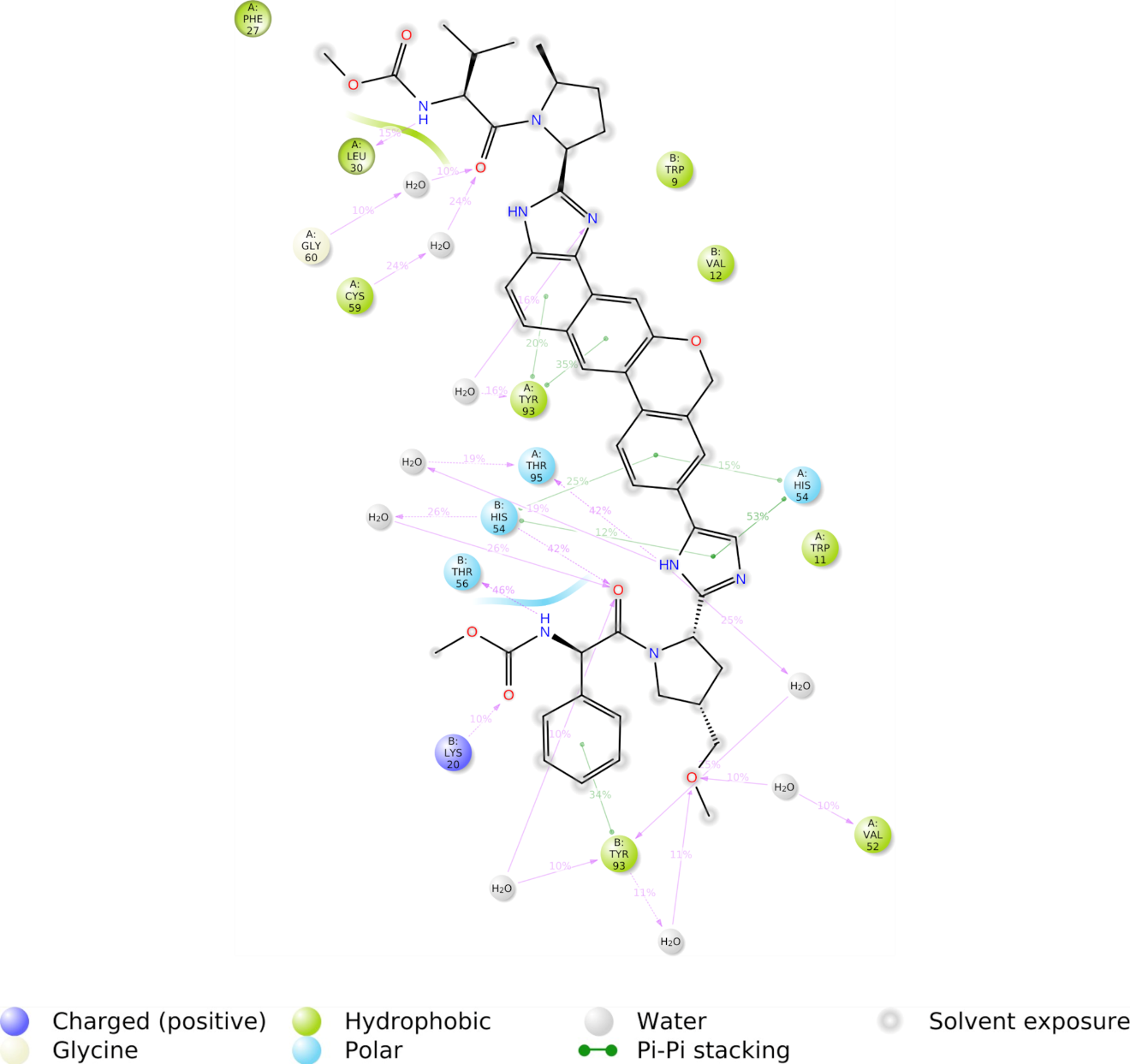


**Fig. S29** Protein-ligand contacts of HCV NS5A GT-4a with velpatasvir


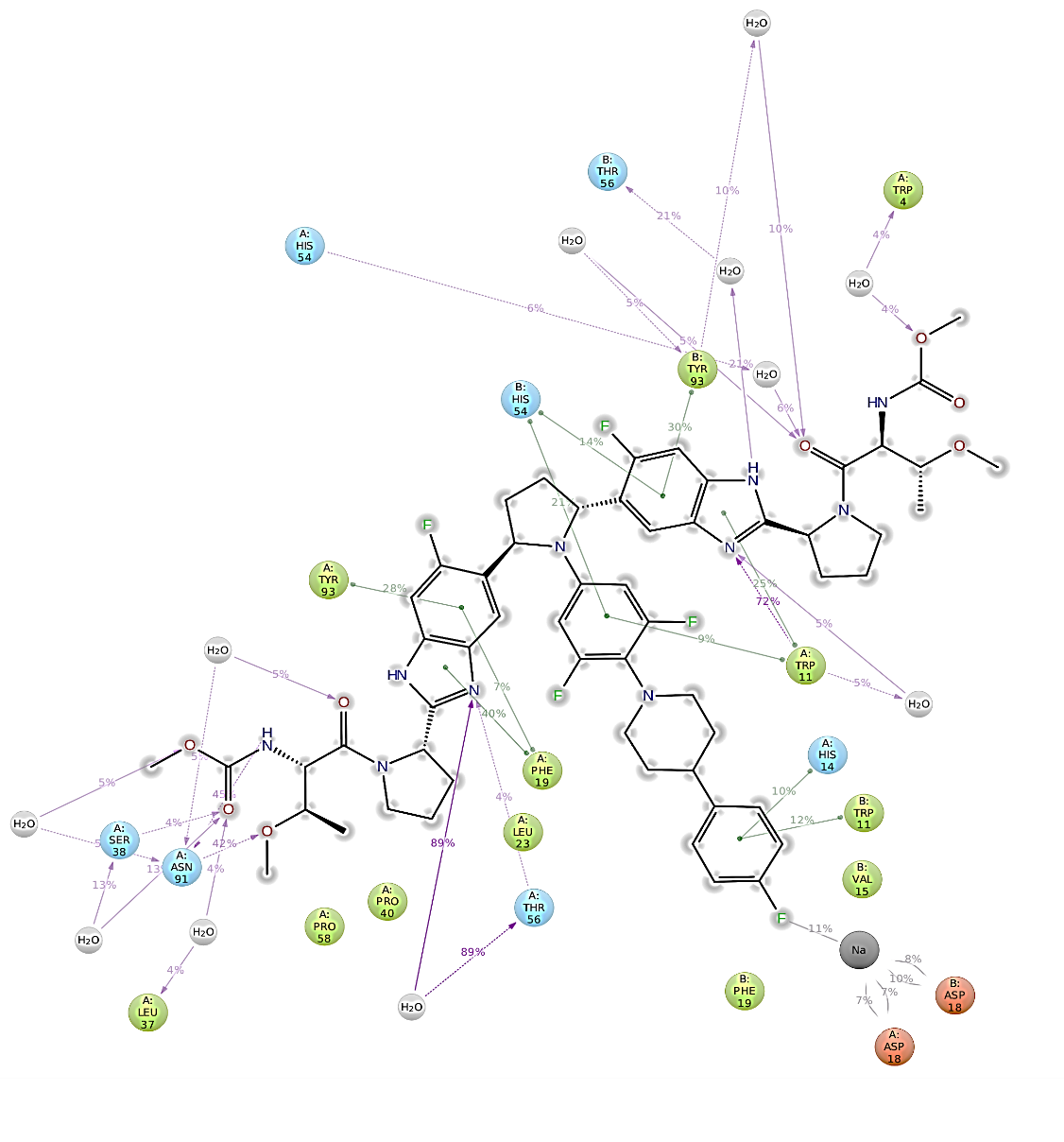

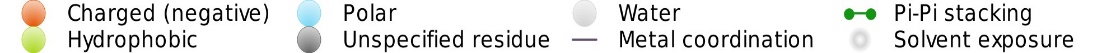


**Fig. S30** Protein-ligand contacts of HCV NS5A GT-4a with pibrentasvir
